# Radiofrequency antenna concepts for human cardiac MR at 14.0 T

**DOI:** 10.1007/s10334-023-01075-1

**Published:** 2023-03-15

**Authors:** Bilguun Nurzed, Andre Kuehne, Christoph Stefan Aigner, Sebastian Schmitter, Thoralf Niendorf, Thomas Wilhelm Eigentler

**Affiliations:** 1 Max-Delbrück-Center for Molecular Medicine in the Helmholtz Association (MDC), Berlin Ultrahigh Field Facility (B.U.F.F.), Robert Rössle Strasse 10, 13125 Berlin, Germany; 2 MRI.TOOLS GmbH, Berlin, Germany; 3 Physikalisch-Technische Bundesanstalt (PTB), Braunschweig, Berlin Germany; 4 Experimental and Clinical Research Center (ECRC), a joint cooperation between the Charité Medical Faculty and the Max-Delbrück-Center for Molecular Medicine in the Helmholtz Association, Berlin, Germany; 5 Chair of Medical Engineering, Technische Universität Berlin, Berlin, Germany

**Keywords:** Electrodynamics, Ultrahigh field MR, Electrical dipole, Parallel transmission, Cardiovascular MRI

## Abstract

**Objective:**

To examine the feasibility of human cardiac MR (CMR) at 14.0 T using high-density radiofrequency (RF) dipole transceiver arrays in conjunction with static and dynamic parallel transmission (pTx).

**Materials and methods:**

RF arrays comprised of self-grounded bow-tie (SGBT) antennas, bow-tie (BT) antennas, or fractionated dipole (FD) antennas were used in this simulation study. Static and dynamic pTx were applied to enhance transmission field (B_1_^+^) uniformity and efficiency in the heart of the human voxel model. B_1_^+^ distribution and maximum specific absorption rate averaged over 10 g tissue (SAR_10g_) were examined at 7.0 T and 14.0 T.

**Results:**

At 14.0 T static pTx revealed a minimum B_1_^+^_ROI_ efficiency of 0.91 μT/√kW (SGBT), 0.73 μT/√kW (BT), and 0.56 μT/√kW (FD) and maximum SAR_10g_ of 4.24 W/kg, 1.45 W/kg, and 2.04 W/kg. Dynamic pTx with 8 kT points indicate a balance between B_1_^+^_ROI_ homogeneity (coefficient of variation < 14%) and efficiency (minimum B_1_^+^_ROI_ > 1.11 µT/√kW) at 14.0 T with a maximum SAR_10g_ < 5.25 W/kg.

**Discussion:**

MRI of the human heart at 14.0 T is feasible from an electrodynamic and theoretical standpoint, provided that multi-channel high-density antennas are arranged accordingly. These findings provide a technical foundation for further explorations into CMR at 14.0 T.

**Supplementary Information:**

The online version contains supplementary material available at 10.1007/s10334-023-01075-1.

## Introduction

The progress of ultrahigh field magnetic resonance (UHF-MR) provides meaningful technologies for advancing biomedical and diagnostic magnetic resonance imaging (MRI). With 7.0 T human MRI now widely used in clinical research, there is increasing interest in exploring even higher magnetic field strengths [1, 2]. This includes pioneering reports on MRI technology at 9.4 T, 10.5 T and 11.7 T, and corresponding in vivo applications [3–12]. The MR research and superconductor science community have already taken even more ambitious steps towards the future, envisioning human MR at 14.0 T [13–16]. Recently, the *Dutch National 14Tesla Initiative in Medical Science* (DYNAMIC) received funding for the implementation of the first 14.0 T class human MR instrument as part of the large-scale research infrastructure national roadmap of the Netherlands [17]. Joint efforts of the nuclear magnetic resonance (NMR) and MRI communities have identified the scientific questions that drive these ambitions, together with the technological challenges and prospects for achieving human MRI at 20.0 T [14–16, 18–21]. These bold steps will require rigorous technical developments, assessment of physiological constraints, and in vivo evaluation studies that have to be tested and validated by those who adopt the technology. Recent experience at 7.0 T offers insights into how such efforts can lead to valuable results [22–27].

Advances in body and cardiovascular magnetic resonance (CMR) imaging at 7.0 T offer a perspective into what we might expect as the technology moves to even higher magnetic field strengths [28, 29]. CMR applications at 7.0 T include imaging and spectroscopy of the heart and large vessels [30, 31]. The spectrum of applications includes high spatial resolution imaging of cardiac morphology and cardiac chamber quantification [32, 33], blood oxygenation level-dependent, susceptibility or iron imaging of the heart [34–37], non-invasive tissue characterization and phenotyping [38], analysis of hemodynamics and heart valve planimetry [39, 40], probing of cardiac energetics [41], computation of myocardial pH [42], and the assessment of myocardial tissue ion concentration including sodium and potassium MRI [43–45]. Clinical CMR at UHF strengths is already conceivable [46–50], though practical and technical issues still need to be resolved before UHF-CMR can move into routine clinical settings [28].

Studies on UHF-CMR are making progress with novel radiofrequency (RF) technologies and MR methodologies to address electrodynamic constraints and transmission field (B_1_^+^) non-uniformities [51–53]. This research includes the implementation of a local transceiver (Tx/Rx) arrays and multi-channel transmission (Tx) arrays in conjunction with multi-channel local receive (Rx) arrays. Surface RF transmit arrays tailored for CMR take advantage of loops [54–57], stripline-configurations [58], stripline waveguide-like elements, slot-antennas [59], dipoles [60], loop-dipoles [61, 62], and building blocks of bow-tie antenna variants [63, 64]. Dipole antenna configurations have received increased attention for UHF-CMR. Dipole antennas provide a symmetrical B_1_^+^ transmission perpendicular to the dipole, which simplifies the optimization of the resulting B_1_^+^ in static pTx [60]. Their linear current patterns help to improve the signal-to-noise ratio (SNR) performance *en route* to ultimate intrinsic SNR [65]. Current dipole antenna array configurations commonly rely on geometric decoupling, which limits the number of Tx elements placed on the torso [60–62].

Multi-channel Tx/Rx RF coil designs tailored for UHF-CMR involve rigid, flexible and modular configurations. The development process has shown a trend towards increasing numbers of transmit and receive elements to improve anatomical coverage. A higher number of RF elements is conceptually appealing to increase the degrees of freedom for B_1_^+^ shaping and uniform B_1_^+^ distribution [66]. A higher channel count benefits signal reception and supports higher acceleration in parallel imaging (PI) [67, 68]. To further highlight Tx array configurations, pioneering work has demonstrated a path towards body coil concepts suited for MR of the torso at 7.0 T [69–73].

Moving to even higher magnetic field strengths, 14.0 T class instruments will facilitate sharper spatiotemporal details of the heart, enable enhanced blood-dependent and tissue contrast mechanisms, and will allow for better and faster visualization of substances relevant to cardiac metabolism.

These opportunities are motivating research into electrodynamics at UHF and are driving innovations in RF antenna design tailored for CMR at frequencies of 600 MHz. Recognizing this, in the current simulation study we present RF coil concepts for human CMR at 14.0 T, and explore the feasibility of multi-element dipole antenna-based RF array configurations. In addition, electromagnetic field (EMF) simulations were conducted in human voxel models to detail B_1_^+^ efficiency (B_1_^+^/√1 kW) and distributions, specific absorption rate (SAR), and PI performance.

## Methods

### RF antenna building blocks

This simulation study builds on dipole variants established for CMR at 7.0 T and MRI of the torso at 10.5 T, including self-grounded bow-tie (SGBT) building blocks [63], bow-tie (BT) building blocks [64] and fractionated dipole (FD) antennas [60–62]. The dimensions of the RF building blocks were adapted to the ^1^H resonance frequency at 14.0 T (f = 600 MHz) and the corresponding wavelength in tissue (~ 5–6 cm). The SGBT has a size of 24.3 × 48.0 × 89.3 mm^3^ at 7.0 T and 12.2 × 24.0 × 44.7 mm^3^ at 14.0 T. For each SGBT a parallel capacitor and a serial inductor were used for tuning and matching. The BT uses a size of 53.0 × 76.0 × 156.0 mm^3^ at 7.0 T and 26.5 × 38.0 × 78.0 mm^3^ at 14.0 T. The tuning and matching circuit consist of a serial and a parallel capacitor. The FD consists of a dipole antenna (7.0 T: 304.0 × 10.0 × 1.6 mm^3^, 14.0 T: 152.0 × 5.0 × 0.8 mm^3^), where low loss optimized meander elements are modeled as lumped elements (7.0 T: L = 33.5 nH, Q = 258.2 at 7.0 T, 14.0 T: L = 17.9 nH, Q = 88.0) between the three segments of the antenna legs. The inductivity was set to minimize the imaginary part of the antennas’ impedance and as a trade-off between superficial SAR and B_1_^+^ [60]. For improved geometric conformity to the upper torso of the human voxel model, a 160° angled FD configuration was used [61]. The tuning and matching circuit consists of a parallel inductor and a serial capacitor, whereas no housing was included for these antenna configurations.

### Cardiac RF arrays

**Fig. 1:**
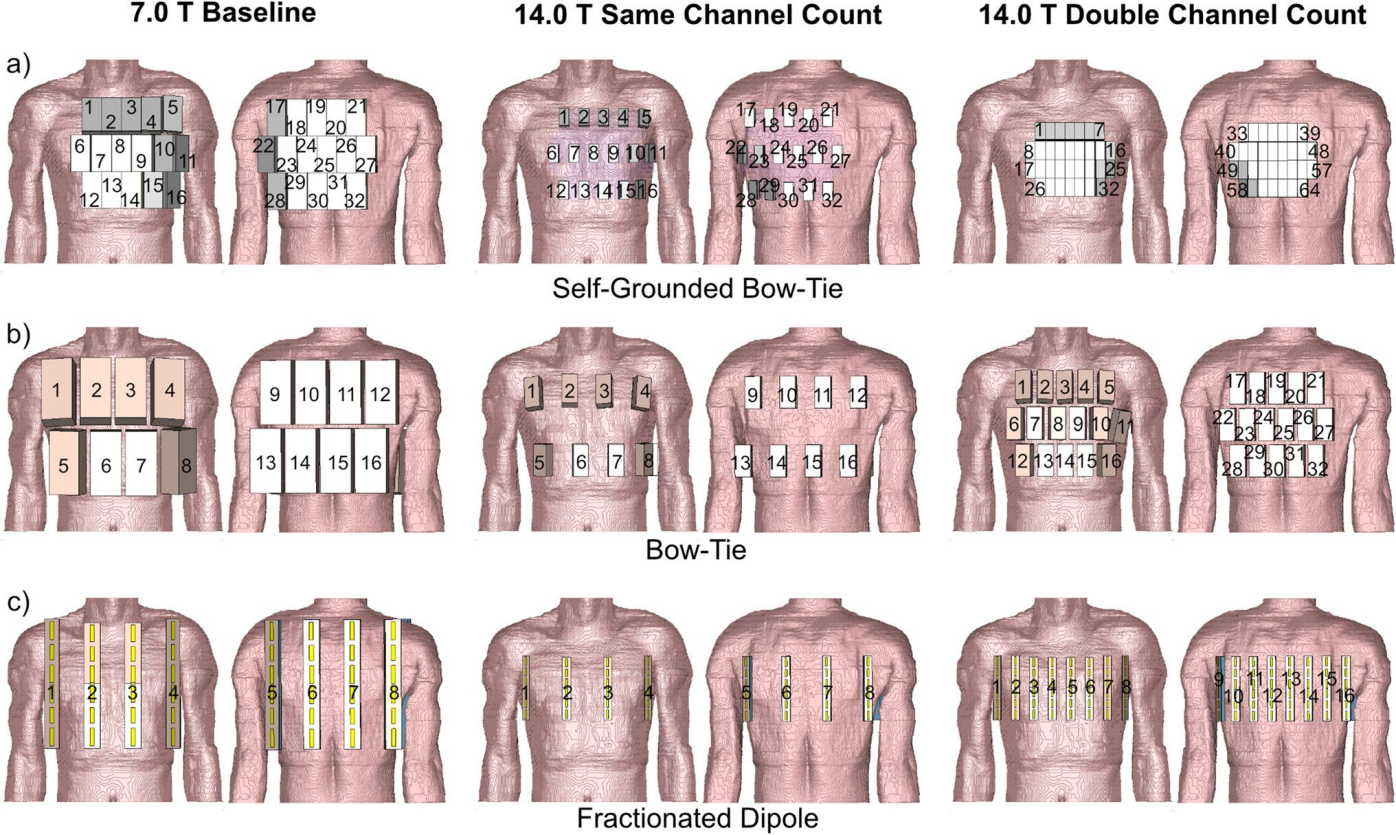
Anterior and posterior views of the cardiac RF arrays using **a** self-grounded bow-tie antenna building blocks; **b** bow-tie antenna building blocks; and **c** fractionated dipole antennas placed on the human voxel model Duke. Duke was truncated at the neck and the hips. For the baseline setups at 7.0 T 32-channel pTx/Rx SGBT, 16-channel pTx/Rx BT, and 8-channel pTx/Rx FD array configurations were used. At 14.0 T the building blocks were assembled into RF arrays with the same numbers, center position and anatomical coverage as the 7.0 T BL setups. These setups are referred as the same channel count setups. For the double channel count setups at 14.0 T the number of building blocks was increased to 64-channel pTx/Rx SGBT, 32-channel pTx/Rx BT, and 16-channel pTx/Rx FD

Three cardiac RF arrays were examined for each building block (BB) (Fig. 1):

- At 7.0 T the BBs were arranged so that each RF array provided ample upper torso coverage (Fig. 1). The BBs were placed with the highest density, resulting in S_ij_ ≤ − 8.6 dB for human voxel Duke and S_ij_ ≤ − 8.3 dB for human voxel model Ella. This setup is referred to as **baseline (BL)**.
- At 14.0 T the BBs were assembled into RF arrays with the number of BBs, the center position of the BBs and the anatomical coverage identical to the setup used at 7.0 T (Fig. 1). This setup is referred to as the **same channel count (SCC)**.
- At 14.0 T the number of BBs was doubled from the 7.0 T setup (Fig. 1). The BBs provided ample upper torso coverage as the 7.0 T BL and 14.0 T SCC setups. This setup is referred to as **double channel count (DCC).**

At 7.0 T BL, a 5–6–5 matrix (anterior and posterior section) of SGBT was used to form a 32-channel parallel transmission (pTx)/Rx RF array (Fig. 1a). No extra space was added between BBs. A 16-channel pTx/Rx RF array (4 × 2 matrix for the anterior and posterior section) was set up for the BT (Fig. 1b). The nearest-neighbor distance was 10 mm. For the FD, an 8-channel pTx/Rx RF array (4 × 1 matrix for the anterior and the posterior section) was used together with a nearest-neighbor distance of 60 mm (Fig. 1c).

At 14.0 T, the SCC setup used the same center position for each BB as implemented at 7.0 T (Fig. 1). The left–right distance between elements was 24.0 mm for the SGBT-based 32-channel pTx/Rx array, 48.0 mm for the BT based 16-channel pTx/Rx array, and 80.0 mm for the FD based 8-channel pTx/Rx array. For the DCC setup at 14.0 T, a 64-channel pTx/Rx SGBT array (7–9–9–7 matrix for the anterior and the posterior section, no additional space between BBs) was used. A 32-channel pTx/Rx array (matrix: 5–6–5 for the anterior and the posterior section, nearest neighbor distance = 10 mm) was examined for the BT. A 16-channel pTx/Rx array (8 × 1 matrix for the anterior and the posterior section, nearest neighbor distance = 25 mm) was investigated for the FD. A dielectric pad consisting of D_2_O was placed between the SGBT RF arrays and the subject to enhance EMF coupling [63]. To conform to the upper torso, the bend FD [61] RF arrays were used for channels 2 and 3 for the BL and the SCC setup, as well as channels 3–6 for the DCC setup. At 14.0 T the FD array was shifted 10 mm towards the feet (z-direction) to ensure full heart coverage (Fig. 1).

### Electromagnetic field simulations

Numerical EMF simulations of the RF arrays were performed using the finite difference time domain solver [74] of CST Studio Suite 2020 (CST Studio Suite 2020, Dassault Systèmes, Vélizy-Villacoublay Cedex, France). Broadband excitation (bandwidth: Δf_ex_ =  ± 50.0 MHz) was applied for a center frequency of f_ex_ = 297.2 MHz and f_ex_ = 600 MHz. The human voxel models Duke (body mass index [BMI] = 23.1 kg/m^2^) and Ella (BMI = 22.7 kg/m^2^) of the Virtual Family (resolution: 1.0 × 1.0 × 1.0 mm^3^) were used [75]. Duke and Ella were truncated at the neck and the hips and placed at the isocenter of an RF shield model of the 7.0 T and 14.0 T MRI bore. For the EMF simulations, the electrical material parameters of the antennas and the tissue parameters provided by the IT ‘IS Foundation [76] were adapted to 297.2 MHz and 600 MHz conditions.

### Co-simulation

For each magnetic field strength, a co-simulation was performed in Matlab 2019b (Mathworks, Natick, MA) for channel-wise tuning and matching with a lossy capacitor and/or a lossy inductor. The estimated losses were evaluated by the equivalent series resistance of the capacitors based on the datasets of non-magnetic ceramic capacitors (atc100c, American Technical Ceramics, NY). The losses of the inductors are considered through the Q-factor according to the database for non-magnetic air-coil inductors (1512sp, Coilcraft Inc., Cary, IL). The results of the EMF simulations and the material/tissue properties were used for the post-processing (Matlab 2019b) to calculate B_1_^+^ and maximum SAR_10g_ distributions at an isotropic resolution of 4.0 × 4.0 × 4.0 mm^3^.

### B_1_ superposition

To benchmark the RF array performance we evaluated the optimal transmit and receive efficiency for each voxel individually. This metric provides a theoretical electromagnetic performance limit [77, 78]. Assessing the RF array transmit efficiency (TXE) and intrinsic SNR (iSNR) requires the B_1_^+^ and B_1_^−^ amplitudes and the power correlation matrix of each RF channel [77]. The loss terms for the RF arrays were evaluated using a framework for calculating the power correlation matrices [79]. The optimal TXE and iSNR are defined by the ratio of the NMR signal (B_1_^+^, B_1_^−^) to the dissipated RF power of the sample. The problem of finding the maximum ratio can be treated as a generalized eigenvalue problem, where the largest eigenvalue corresponds to the maximum TXE and iSNR [77, 78]. For the intrinsic optimal magnitude superposition of the B_1_^+^ and B_1_^−^ fields only the sample losses are considered, and for the realistic superposition sample, coil and coupling losses are taken into account. The ratio between intrinsic and realistic B_1_^+^ and B_1_^−^ superposition is defined as the performance ratio (%). The calculated TXE and iSNR maps are assessed and compared within the region of interest (ROI) covering the entire 3D heart.

### Field shaping for static parallel transmission

The optimization was based on the magnitude of the sum of the complex B_1_^+^ maps in the ROI covering the entire 3D heart, with a channel-specific normalized complex excitation vector *exc*_*ch*_ (*exc*_*ch*_*/*abs(*exc*_*ch*_)) [80]. Field shaping was first performed for static pTx to determine an optimal *exc*_*ch*_ using channel-wise RF phase optimization or channel-wise RF phase and RF amplitude optimization. The transmission field-shaping was performed using an unconstrained genetic algorithm (GA) in combination with an unconstrained minimization (fminunc) implemented in the global optimization toolbox of Matlab 2019b [81, 82]. The total RF power for the excitation vectors (*P*_*fwd*_) obtained from the pTx field shaping can be calculated following the equation:

1 $${P}_{fwd}={ex{c}^{H} \cdot (I}_{ch}\cdot \frac{{U}^{2}}{R})\cdot exc$$

where superscript *H* denotes conjugate transpose, *exc* the complex excitation vector for *N*_*ch*_ channels, *I*_*ch*_ the identity matrix for *N*_*ch*_ channels, with *R* = 50 Ω and *U*≈316 V if we consider 2 kW at each port without losses. The obtained B_1_^+^ maps of the optimization were scaled to the root mean square of 1 kW as a total incident power *P*_*In*_ (power flow into ports) which is referred to as B_1_^+^ efficiency (B_1_^+^ /√1 kW).

#### Minimum B_1_^+^ optimization

To avoid signal dropouts the minimum of the superposed B_1_^+^ of the individual channels (Eq. 2) across the ROI covering the entire 3D heart was maximized using the target function:

2 $$Maximize {\Phi }_{target}\left({\mathrm{exc}}_{\mathrm{ch}}\right)=\mathrm{ min}\left({\left|{\sum }_{ch=1}^{{N}_{ch}}{{B}_{1}^{+}}_{ch}\cdot {exc}_{ch}\right|}_{ROI}\right)$$

with *N*_*ch*_ being the number of channels, *B*_*1*_^+^_*ch*_ the channel-wise complex transmission field inside the 3D ROI, and *exc*_*ch*_ the complex excitation vector for *N*_*ch*_ channels.

#### Coefficient of variation optimization

To minimize the coefficient of variation (CoV = standard deviation/mean) across 3D ROI covering the entire heart, the following target function was used:

3 $$Minimize {\Phi }_{target}\left({\mathrm{exc}}_{\mathrm{ch}}\right)=(\frac{\mathrm{SD}\left({\left|{\sum }_{ch=1}^{{N}_{ch}}{{B}_{1}^{+}}_{ch}\cdot {exc}_{ch}\right|}_{ROI }\right)}{\mathrm{mean}\left({\left|{\sum }_{ch=1}^{{N}_{ch}}{{B}_{1}^{+}}_{ch}\cdot {exc}_{ch}\right|}_{ROI }\right)})$$

The coefficient of variation indicates the (non)uniformity of the B_1_^+^ distribution.

#### SAR optimization

A multiobjective optimizer (MOO) is used to perform a trade-off between two objectives using the GA [82]. The resulting Pareto-front of the MOO finds a solution in which one objective is improved and one objective degraded. For better SAR management at higher static magnetic field strength, SAR is included as one of the objectives, and minimum B_1_^+^_ROI_ as the other objective in the MOO approach. SAR_10g_ distribution was compressed using virtual observation points (VOP) [83]. The overestimation factor for the VOP calculation was iteratively reduced until reaching a mean overestimation of 15%. The VOP with a mean overestimation of 15% was only used in the optimization process. The number of VOP was at 7.0 T < 1493 and at 14.0 T with double the channel count < 23,579.

The target function ${\Phi }_{totat}$= *(*${\Phi }_{target},{\Phi }_{SAR})$ is given by:

4 $${Minimize \Phi }_{total}\left({\mathrm{exc}}_{\mathrm{ch}}\right)=(-\mathrm{min}\left({\left|{\sum }_{ch=1}^{{N}_{ch}}{{B}_{1}^{+}}_{ch}\cdot {exc}_{ch}\right|}_{ROI }\right), \mathrm{ max}({{exc}_{ch}}^{H} \cdot VOP\cdot {exc}_{ch}))$$

where superscript *H* denotes conjugate transpose. To maximize the minimum B_1_^+^_ROI_ in this minimization approach a minus sign was added for the target function. From the results of the MOO, the non-compressed SAR matrix was used for each excitation vector of the solution. Based on the results an excitation vector maximizing (minimum B_1_^+^_ROI_/√SAR_10g_) was evaluated.

### Field shaping with dynamic parallel transmission

Dynamic pTx was performed with tailored kT-points, a series of RF sub-pulses and gradient blips, with the goal of 3D flip angle (FA) homogenization (CoV(FA)) targeting the whole heart [84]. The pulse design problem [52] was solved in Matlab 2019b using the small-tip-angle approximation (STA) for a nominal FA distribution of 10° across the whole heart with an interleaved greedy + local method [52, 85, 86]. The computation of the solution included a global RF power regularization but no local SAR constraints. 4 and 8 kT point pTx pulses were optimized with rectangular-shaped RF sub-pulses and a total pulse duration of τ_total_ = 0.96 ms (4 × τ_sub-pulse_ = 100 µs, 4 × τ_blips_ 140 µs) and τ_total_ = 1.92 ms (8 × τ_sub-pulse_ = 100 µs, 8 × τ_blips_ 140 µs), respectively.

5 $$\begin{gathered} B_{1eff}^{ + } = \frac{FA}{{2\pi \gamma \tau_{sub - pulse} }} \cdot \frac{{\sqrt {P_{In} } }}{{\sqrt {P_{fwd} \cdot k} }} \hfill \\ k = \frac{{\tau_{total} }}{1ms} \cdot \frac{{\tau_{sub - pulse} + \tau_{blip} }}{{\tau_{sub - pulse} }} \hfill \\ \end{gathered}$$

where *γ* denotes the gyromagnetic ratio, P_fwd_ the forward power and *k* the power scaling factor. The pulse duration of the kT point pTx pulses was scaled to 1 ms for an inserted power (P_In_) of 1 kW to compare dynamic and static pTx approaches. The obtained FA maps (*FA* = *γ B*_*1*_^+^ *τ)* were scaled into B_1_^+^ efficiency maps where the forward power (P_fwd_) of the kT points was scaled to 1 ms ($\frac{{\tau }_{total}}{1ms}$) and only the time of the sub-pulses ($\frac{{\tau }_{sub-pulse}+{\tau }_{blip}}{{\tau }_{subpulse}})$ was considered. The maximum SAR_10g_ (P_In_ = 1W) of the kT points was evaluated from the sum of the SAR_10g_ distribution for each sub-pulse.

### Assessment of noise amplification (G-factor)

A post-processing framework was used to assess the parallel imaging (PI) performance through SENSE geometry (g) factor maps [67, 68]. The maps were calculated using reduction factors of R = 2 to R = 4. The phase encoding (PE) direction was placed along the main left–right (L–R, y-axis) and along the semi-minor anterior–posterior (A–P, x-axis) direction. G-factor assessment was performed for 1D SENSE acceleration using field of view (FOV) = 324 × 232 mm (matrix size: 81 × 58, voxel size 4.0 × 4.0 × 4.0 mm^3^) for an axial (x–y) plane through the center of the heart of the voxel model.

## Results

### Co-simulation

The worst-case reflection and coupling for Duke and Ella after tuning and matching can be found in Table 1. The SGBT tuning and matching network was model specific, and showed a high deviation between Duke and Ella for the given setup. For all setups, C values (min.-max.) of 0.2 pF–31.7 pF (Duke) and 0.2 pF–19.0 pF (Ella) were found. The L values (min.-max.) were 2.5 nH–20.2 nH (Duke) and 2.5 nH–18.4 nH (Ella). The BT tuning and matching network was robust against different models and showed minor deviation between Duke and Ella. The serial C values were between 1.8 pF–7.7 pF (Duke) and 1.9 pF–6.6 pF (Ella) whereas the parallel C values were between 2.8 pF–14.8 pF (Duke) and 3.3 pF–14.3 pF (Ella). The FD tuning and matching network was model specific, with a high deviation between Duke and Ella for a given setup. L values of (min.–max.) of 12.2 nH–61.4 nH (Duke) and 16.8 nH–62.0 nH (Ella) were found and C values (min.–max.) of 3.8 pF–9.7 nF (Duke) and 3.1 pF–1.60 nF (Ella) were found.

**Table 1:** Simulated maximum reflection (S_ii_) and coupling (S_ij_) values after tuning and matching for the 7.0 T baseline (BL), 14.0 T same channel count (SCC), and double channel count (DCC) setups using self-grounded bow-tie (SGBT) antenna building blocks, bow-tie (BT) antenna building blocks, and fractionated dipole (FD) antennas placed on the human voxel models Duke and Ella

Antenna	max dB	7.0 T BL	14.0 T SCC	14.0 T DCC
Simulated maximum reflection (S_ii_) and coupling (S_ij_) for Duke				
SGBT	Reflection S_ii_	− 27.5	− 63.5	− 18.9
	Coupling S_ij_	− 9.4	− 20.0	− 10.6
BT	Reflection S_ii_	− 21.2	− 44.9	− 24.4
	Coupling S_ij_	− 8.6	− 14.5	− 9.8
FD	Reflection S_ii_	− 21.4	− 47.2	− 25.4
	Coupling S_ij_	− 15.3	− 15.7	− 10.3
Simulated maximum reflection (S_ii_) and coupling (S_ij_) for Ella				
SGBT	Reflection S_ii_	− 17.7	− 23.5	− 12.7
	Coupling S_ij_	− 8.5	− 13.7	− 10.2
BT	Reflection S_ii_	− 22.2	− 45.2	− 25.4
	Coupling S_ij_	− 8.3	− 15.1	− 9.7
FD	Reflection S_ii_	− 29.9	− 15.3	− 21.1
	Coupling S_ij_	− 13.0	− 14.9	− 8.4

### B_1_ superposition

**Fig. 2:**
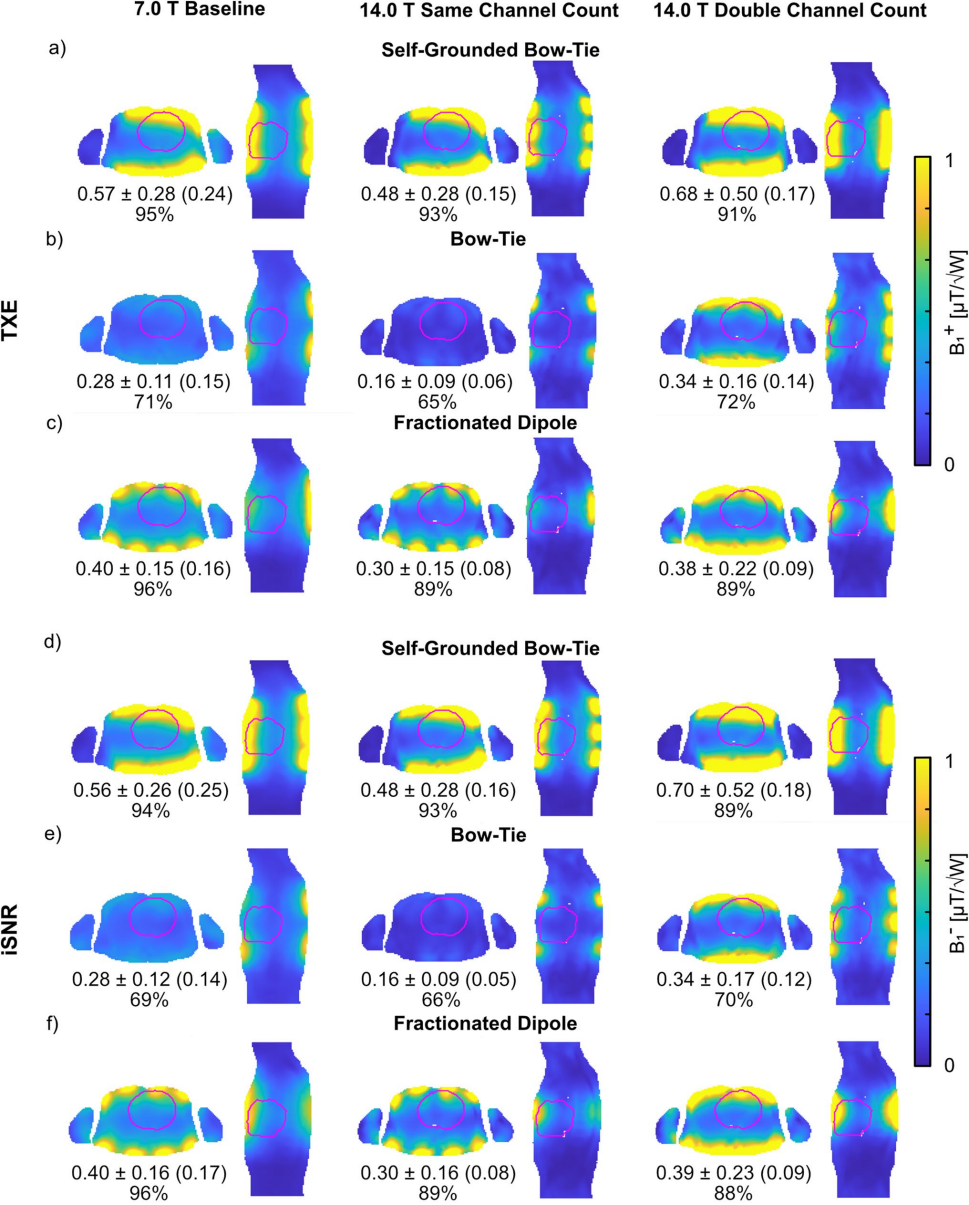
Axial and sagittal views through the center of the heart ROI showing realistic **a**–**c** B_1_^+^ (TXE) and **d**–**f** B_1_^−^ (iSNR) superposition maps (accounting for sample, coil, and coupling losses). Annotations highlight the mean ± SD (minimum) TXE and iSNR values and the mean performance ratio in % over the whole 3D cardiac ROI using the a, d) self-grounded bow-tie antenna building block; b, e) bow-tie antenna building block; and c,f) the fractionated dipole antenna RF arrays at 7.0 T (baseline) and 14.0 T (same channel count and double channel count). The cardiac ROI is depicted in red

The sum of the magnitude of the superposed B_1_^+^ (Fig. 2) revealed a lower TXE (realistic) for Duke at 14.0 T with the SCC setups compared to the 7.0 T BL setups, where the BT array showed the largest decrease in the mean value of − 43% and the SGBT showed the smallest decrease in the mean value of − 16%. Increasing the channel count for the DCC setups at 14.0 T revealed in the best-case 113% higher mean and 130% higher minimum TXE (realistic) for the BT array, and in the worst-case 26% higher mean and 13% higher minimum TXE (realistic) for the FD array, compared to the SCC setups. The DCC setups had the largest standard deviation of the RF array configurations investigated. The DCC setups had increased mean TXE for the BT (+ 21%) and SGBT (+ 19%), relative to the 7.0 T BL setups, but decreased mean TXE for the FD (− 5%) as well as decreased minimum values. The iSNR values for reception are shown in Fig. 2. Similar behavior could be obtained for Ella with only higher TXE/iSNR for a given setup (data not shown).

### Field shaping using static pTx 

PTx using an excitation vector with equal phase (0°) and amplitude (1) for all channels was used as a baseline (Table 2). The baseline pTx provided for Duke a minimum B_1_^+^_ROI_ < 0.05 µT/√kW, a CoV < 56% for an ROI covering the entire heart, and a maximum SAR_10g_ < 0.67 W/kg for all RF arrays at 7.0 T (BL) and 14.0 T (SCC and DCC) (Table 2). The baseline pTx results for Ella are shown in Table 2.

**Table 2:** Summary of the mean B_1_^+^, minimum B_1_^+^, coefficient of variation (CoV(B_1_^+^_ROI_)) across the entire 3D heart of the human voxel models Duke and Ella, and the maximum SAR_10g_ for an excitation vector with equal phase (0°) and amplitude (1 V) for all channels using self-grounded bow-tie (SGBT) antenna building block, bow-tie (BT) antenna building block, and fractionated dipole (FD) antenna RF arrays at 7.0 T (baseline, BL) and 14.0 T (same channel count, SCC, double channel count, DCC). The total RF power for the excitation vectors (P_fwd_) is presented for a lossless 2 kW power at each channel

	Excitation with equal phase (0°) and amplitude (1)				
	mean B_1_^+^_ROI_ [µT/√kW]	min. B_1_^+^_ROI_ [µT/√kW]	max. SAR_10g_ [W/kg]	CoV [%]	P_fwd_ [kW]
Duke					
7.0 T BL					
SGBT	5.21	0.02	0.30	45	64
BT	3.40	0.03	0.23	44	32
FD	5.05	0.01	0.25	39	16
14.0 T SCC					
SGBT	3.37	0.05	0.62	52	64
BT	1.96	0.01	0.67	55	32
FD	3.38	0.04	0.57	48	16
14.0 T DCC					
SGBT	4.58	0.03	0.45	56	128
BT	2.80	0.03	0.25	46	64
FD	2.51	0.03	0.41	48	32
Ella					
7.0 T BL					
SGBT	5.41	0.06	0.29	39	64
BT	4.31	0.04	0.19	41	32
FD	6.17	0.06	0.28	39	16
14.0 T SCC					
SGBT	3.70	0.01	1.08	51	64
BT	2.44	0.02	0.50	44	32
FD	3.51	0.02	0.40	41	16
14.0 T DCC					
SGBT	4.85	0.03	0.49	52	128
BT	3.21	0.04	0.22	39	64
FD	2.79	0.03	0.27	42	32

#### Minimum B_1_^+^ optimization

**Fig. 3:**
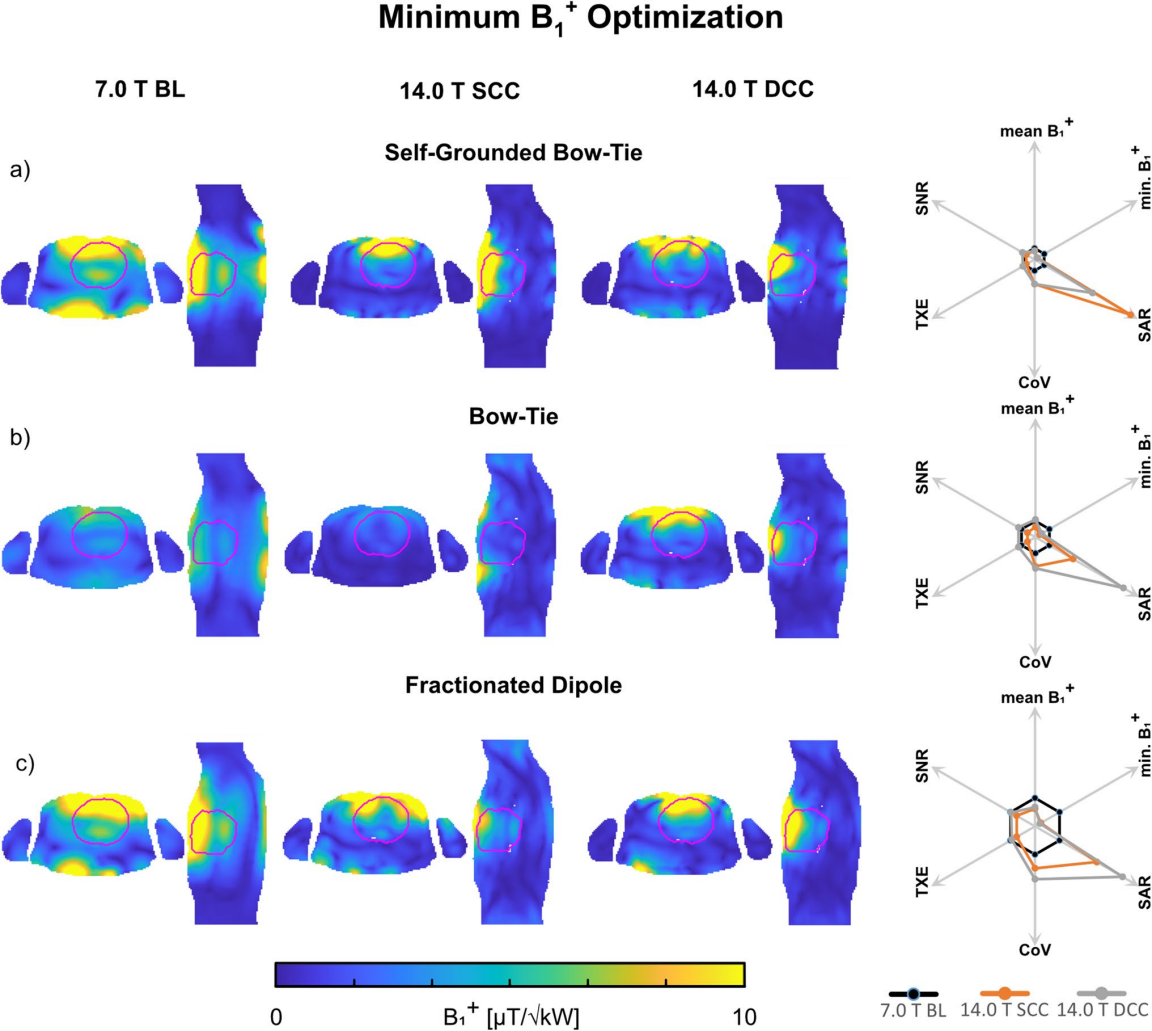
Axial and sagittal views through the center of the heart showing B_1_^+^ efficiency maps (B_1_^+^/√1 kW) obtained for static pTx phase and amplitude shimming using the **a** self-grounded bow-tie antenna building block; **b** bow-tie antenna building block; and **c** the fractionated dipole antenna RF array configurations at 7.0 T (baseline, BL) and 14.0 T (same channel count, SCC, double channel count, DCC). The cardiac ROI is depicted in red. The superposed minimum B_1_^+^ of all channels within the whole 3D cardiac ROI was maximized in the optimization process. The spider diagrams illustrate the relative changes of the mean B_1_^+^_ROI_, minimum B_1_^+^_ROI_, maximum SAR_10g_, CoV(B_1_^+^_ROI_), TXE, and intrinsic SNR values for the 14.0 T SCC (orange) and DCC (grey) with respect to the 7.0 T baseline (black)

For Duke, phase and amplitude optimized pTx had higher minimum B_1_^+^_ROI_ > 1.59 µT/√kW for the 7.0 T BL setups (Fig. 3). At 14.0 T, the SCC setups had ~ 72% lower minimum B_1_^+^_ROI_ compared to the 7.0 T BL setups (Table 3). Increasing the channel count for the DCC setups at 14.0 T resulted in a 46% increased minimum B_1_^+^_ROI_ only for the BT setup, but with a higher SAR level. The SGBT and FD showed 10–15% lower minimum B_1_^+^_ROI_ whereas only the SGBT showed a lower SAR level. The phase and amplitude optimized pTx approach resulted in elevated CoV values. The corresponding results for Ella can be obtained at the bottom in Table 3.

**Table 3:** Summary of the mean B_1_^+^, minimum B_1_^+^, coefficient of variation (CoV(B_1_^+^_ROI_)) across the entire 3D heart of the human voxel models Duke and Ella, and the maximum SAR_10g_ for a phase and amplitude pTx approach with optimized minimum B_1_^+^ in the ROI using self-grounded bow-tie (SGBT) antenna building block, bow-tie (BT) antenna building block, and fractionated dipole (FD) antenna RF arrays at 7.0 T (baseline, BL) and 14.0 T (same channel count, SCC, double channel count, DCC). The total RF power for the excitation vectors (P_fwd_) is presented for a lossless 2 kW power at each channel

	Minimum B_1_^+^ optimization				
	mean B_1_^+^_ROI_ [µT/√kW]	min. B_1_^+^_ROI_ [µT/√kW]	max. SAR_10g_ [W/kg]	CoV [%]	P_fwd_ [kW]
Duke					
7.0 T BL					
SGBT	6.82	3.32	0.71	41	16
BT	3.50	1.59	0.23	36	14
FD	7.44	2.81	0.57	42	5
14.0 T SCC					
SGBT	5.37	1.01	7.01	90	3
BT	2.25	0.50	0.62	66	5
FD	4.61	0.66	1.44	62	5
14.0 T DCC					
SGBT	5.01	0.91	4.24	90	8
BT	3.85	0.73	1.45	70	5
FD	5.05	0.56	2.04	78	4
Ella					
7.0 T BL					
SGBT	7.50	4.59	0.67	34	13
BT	4.48	2.31	0.17	31	8
FD	8.81	4.55	0.63	37	5
14.0 T SCC					
SGBT	6.71	1.43	5.73	89	3
BT	2.26	0.63	0.65	44	5
FD	5.41	0.96	2.44	74	2
14.0 T DCC					
SGBT	6.49	1.64	3.61	74	8
BT	4.45	1.30	1.05	65	7
FD	5.41	1.00	1.44	71	4

#### Coefficient of variation optimization

**Fig. 4:**
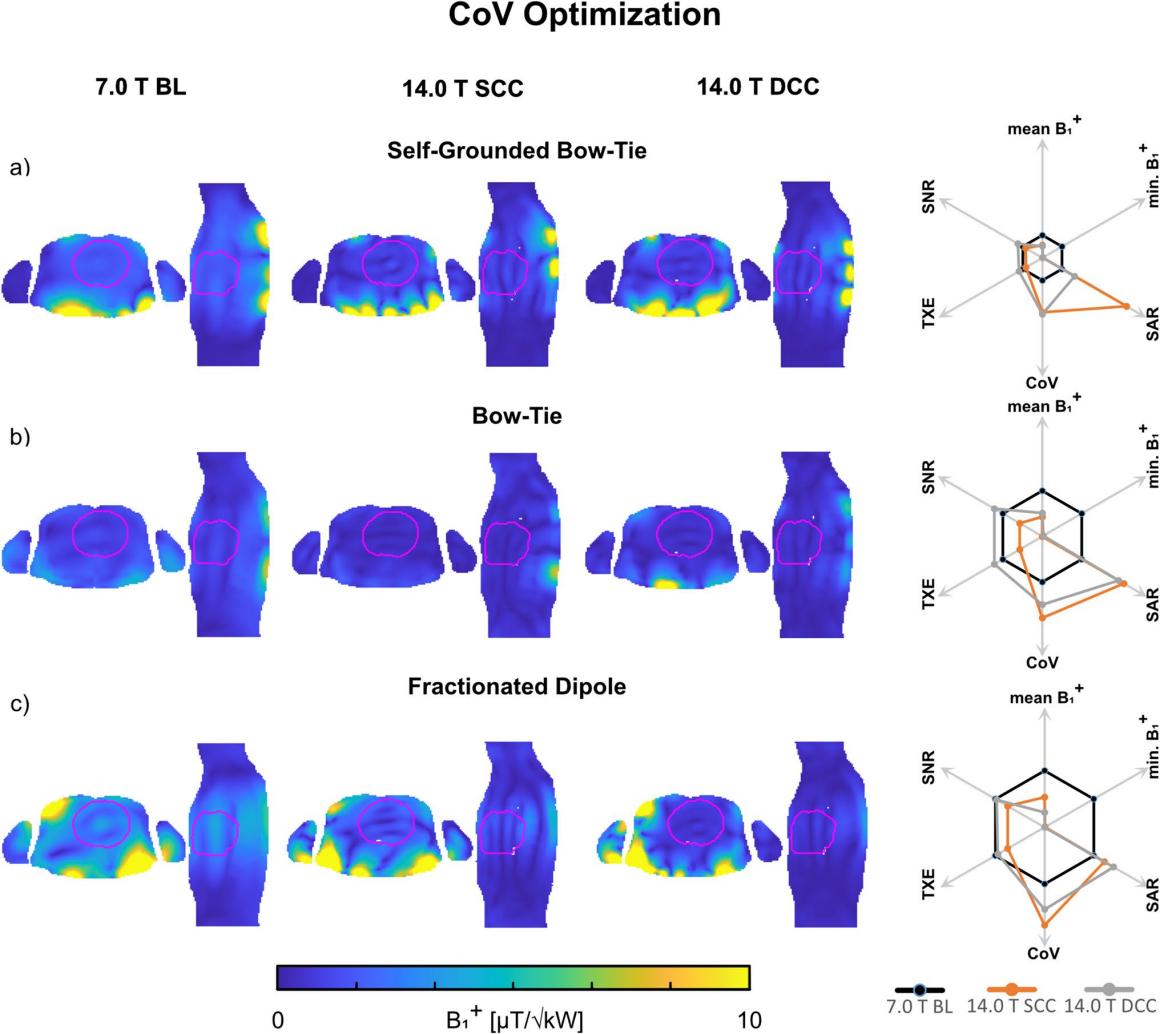
Axial and sagittal views through the center of the heart showing B_1_^+^ efficiency maps (B_1_^+^/√1 kW) obtained for static pTx phase and amplitude shimming using the **a** self-grounded bow-tie antenna building block; **b** bow-tie antenna building block; and **c** the fractionated dipole antenna RF array configurations at 7.0 T (baseline, BL) and 14.0 T (same channel count, SCC, double channel count, DCC). The cardiac ROI is depicted in red. The CoV (B_1_^+^) within the whole 3D cardiac ROI was minimized in the optimization process. The spider diagrams illustrate the relative changes of the mean B_1_^+^_ROI_, minimum B_1_^+^_ROI_, maximum SAR_10g_, CoV(B_1_^+^_ROI_), TXE, and intrinsic SNR values for the 14.0 T SCC (orange) and DCC (grey) with respect to the 7.0 T baseline (black)

For Duke, phase and amplitude optimized pTx showed at least a two-fold decrease in the CoV for minimized CoV(B_1_^+^_ROI_) (Fig. 4) with an elevated minimum B_1_^+^_ROI_ > 0.37 µT/√kW for the 7.0 T BL setups compared to the baseline pTx with equal excitation. The 14.0 T SCC setups had a CoV < 35% with a lower minimum B_1_^+^_ROI_ < 0.01 µT/√kW and a high SAR level < 7.09 W/kg (Table 4). The DCC setups demonstrated a further decreased CoV < 29% with a minimum B_1_^+^_ROI_ < 0.02 µT/√kW and a SAR level < 2.71 W/kg. The corresponding results for Ella are shown in Table 4.

**Table 4:** Summary of the mean B_1_^+^, minimum B_1_^+^, coefficient of variation (CoV(B_1_^+^_ROI_)) across the entire heart of the human voxel models Duke and Ella, and the maximum SAR_10g_ for a phase and amplitude pTx approach with optimized CoV(B_1_^+^) in the ROI using self-grounded bow-tie (SGBT) antenna building block, bow-tie (BT) antenna building block, and fractionated dipole (FD) antenna RF arrays at 7.0 T (baseline, BL) and 14.0 T (same channel count, SCC, double channel count, DCC). The total RF power for the excitation vectors (P_fwd_) is presented for a lossless 2 kW power at each channel

	CoV optimization				
	mean B_1_^+^_ROI_ [µT/√kW]	min. B_1_^+^_ROI_ [µT/√kW]	max. SAR_10g_ [W/kg]	CoV [%]	P_fwd_ [kW]
Duke					
7.0 T BL					
SGBT	1.64	0.88	1.65	10	9
BT	0.96	0.37	0.30	18	6
FD	2.48	1.17	1.45	20	3
14.0 T SCC					
SGBT	0.92	0.01	7.09	25	6
BT	0.41	0.00	0.62	32	6
FD	1.32	0.01	1.76	35	5
14.0 T DCC					
SGBT	0.85	0.02	2.71	26	11
BT	0.49	0.01	0.58	27	7
FD	0.65	0.00	2.03	29	5
Ella					
7.0 T BL					
SGBT	1.89	1.01	3.09	14	5
BT	1.71	0.88	0.64	15	5
FD	3.27	1.63	1.19	16	4
14.0 T SCC					
SGBT	1.34	0.05	2.45	21	13
BT	0.59	0.03	1.45	27	7
FD	1.51	0.07	2.95	28	3
14.0 T DCC					
SGBT	1.10	0.10	2.50	22	16
BT	1.27	0.01	0.61	24	19
FD	1.24	0.03	1.36	24	8

#### SAR optimization

**Fig. 5:**
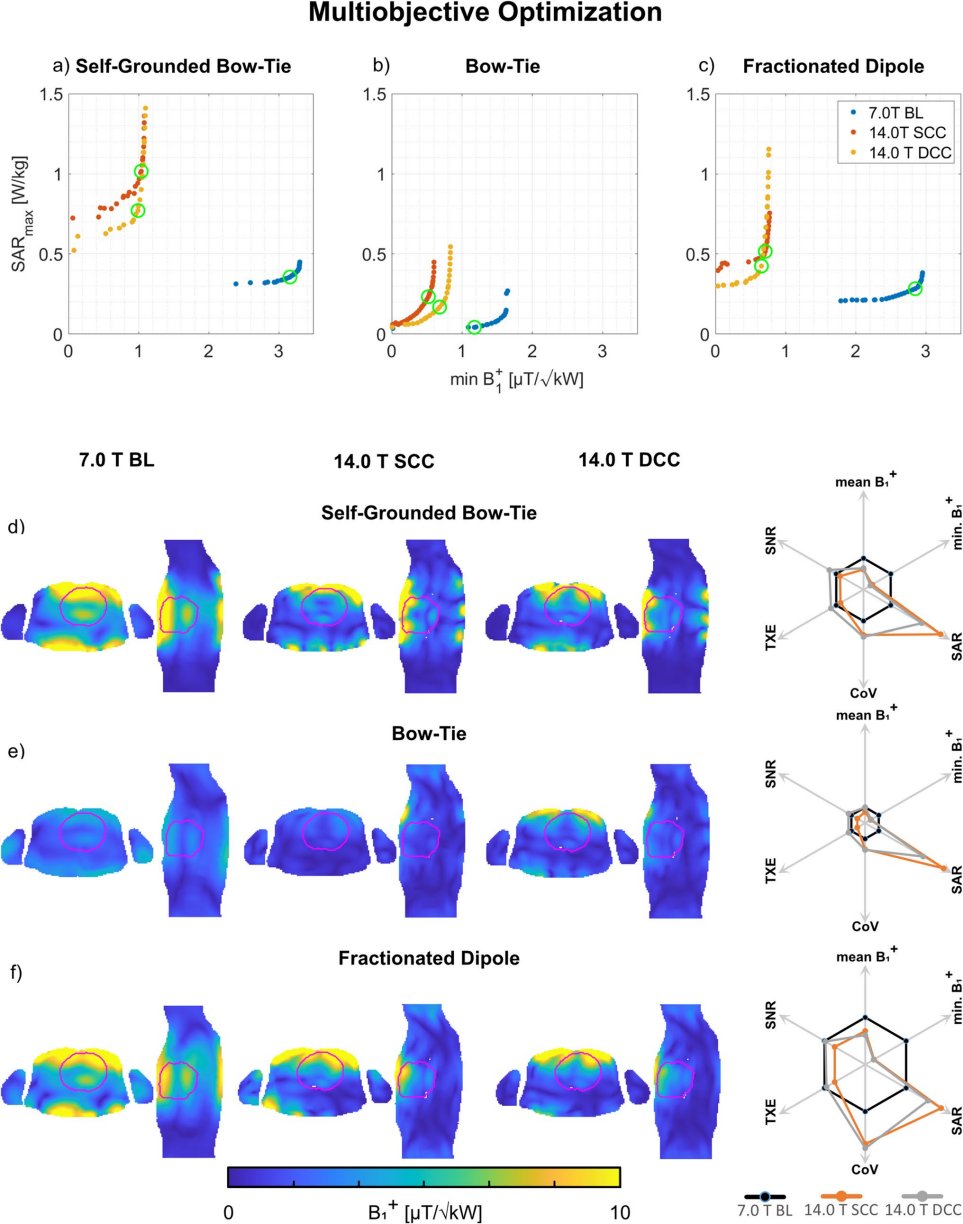
**a**–**c** Pareto front derived from the static phase and amplitude optimized pTx MOO approach using the **a** self-grounded bow-tie antenna building block; **b** bow-tie antenna building block; and **c** the fractionated dipole antenna RF array configurations at 7.0 T (baseline, BL) and 14.0 T (same channel count, SCC, double channel count, DCC). Each point of the solution represents one optimized excitation vector where a trade-off between the minimum B_1_^+^_ROI_ and the maximum SAR_10g_ was found. The green circles indicate the highest minimum B_1_^+^_ROI_/√SAR_10g_ ratio. **d**–**f** Axial and sagittal views through the center of the heart (depicted in red) illustrating B_1_^+^ efficiency maps (B_1_^+^/√1 kW) obtained for the excitation vectors with the highest minimum B_1_^+^_ROI_/√SAR_10g_ ratio (indicated by the green circles in a-c). The spider diagrams illustrate the relative changes of the mean B_1_^+^_ROI_, minimum B_1_^+^_ROI_, maximum SAR_10g_, CoV(B_1_^+^_ROI_), TXE, and intrinsic SNR values for the 14.0 T SCC (orange) and DCC (grey) with respect to the 7.0 T baseline (black)

Moving towards 14.0 T revealed an increased SAR level which was addressed by the phase and amplitude pTx optimized MOO approach (Fig. 5). For Duke, the 14.0 T SCC setups were capable of 63–85% reduction in maximum SAR_10g_ with only 6–11% reduction in minimum B_1_^+^_ROI_ (Table 5) compared to the static pTx approach with maximized minimum B_1_^+^_ROI_ (Table 3, SCC setups). The DCC setups with increased channel count had 79–88% reduced maximum SAR_10g_ with only 0–29% reduced minimum B_1_^+^_ROI_ (Table 5) compared to the static pTx approach with maximized minimum B_1_^+^_ROI_ (Table 3, DCC setups). The MOO revealed a CoV above 54% at 14.0 T for both setups. The corresponding results for Ella are shown in Table 5.

**Table 5:** Summary of the mean B_1_^+^, minimum B_1_^+^, coefficient of variation (CoV(B_1_^+^_ROI_)) across the entire heart of the human voxel models Duke and Ella, and the maximum SAR_10g_ for the multiobjective phase and amplitude optimizer, with a trade-off between minimum B_1_^+^ in the heart and maximum SAR_10g_ using self-grounded bow-tie (SGBT) antenna building block, bow-tie (BT) antenna building block, and fractionated dipole (FD) antenna RF arrays at 7.0 T (baseline, BL) and 14.0 T (same channel count, SCC, double channel count, DCC). The total RF power for the excitation vectors (P_fwd_) is presented for a lossless 2 kW power at each channel

	Multiobjective optimization				
	mean B_1_^+^_ROI_ [µT/√kW]	min. B_1_^+^_ROI_ [µT/√kW]	max. SAR_10g_ [W/kg]	CoV [%]	P_fwd_ [kW]
Duke					
7.0 T BL					
SGBT	6.76	2.84	0.36	38	21
BT	2.53	1.16	0.04	32	8
FD	6.18	2.76	0.28	33	9
14.0 T SCC					
SGBT	4.32	0.91	1.02	55	21
BT	1.80	0.47	0.23	54	13
FD	4.39	0.59	0.52	56	8
14.0 T DCC					
SGBT	4.63	0.73	0.77	57	27
BT	2.54	0.52	0.17	55	13
FD	3.86	0.56	0.43	59	8
Ella					
7.0 T BL					
SGBT	6.96	4.29	0.28	26	20
BT	3.80	1.95	0.10	28	8
FD	7.34	3.88	0.24	27	8
14.0 T SCC					
SGBT	5.60	1.44	1.85	65	8
BT	2.38	0.48	0.21	41	7
FD	4.82	0.98	0.53	54	6
14.0 T DCC					
SGBT	5.71	1.51	0.81	58	20
BT	3.04	0.94	0.22	48	11
FD	3.79	0.88	0.39	59	8

### Field shaping using dynamic pTx

**Fig. 6:**
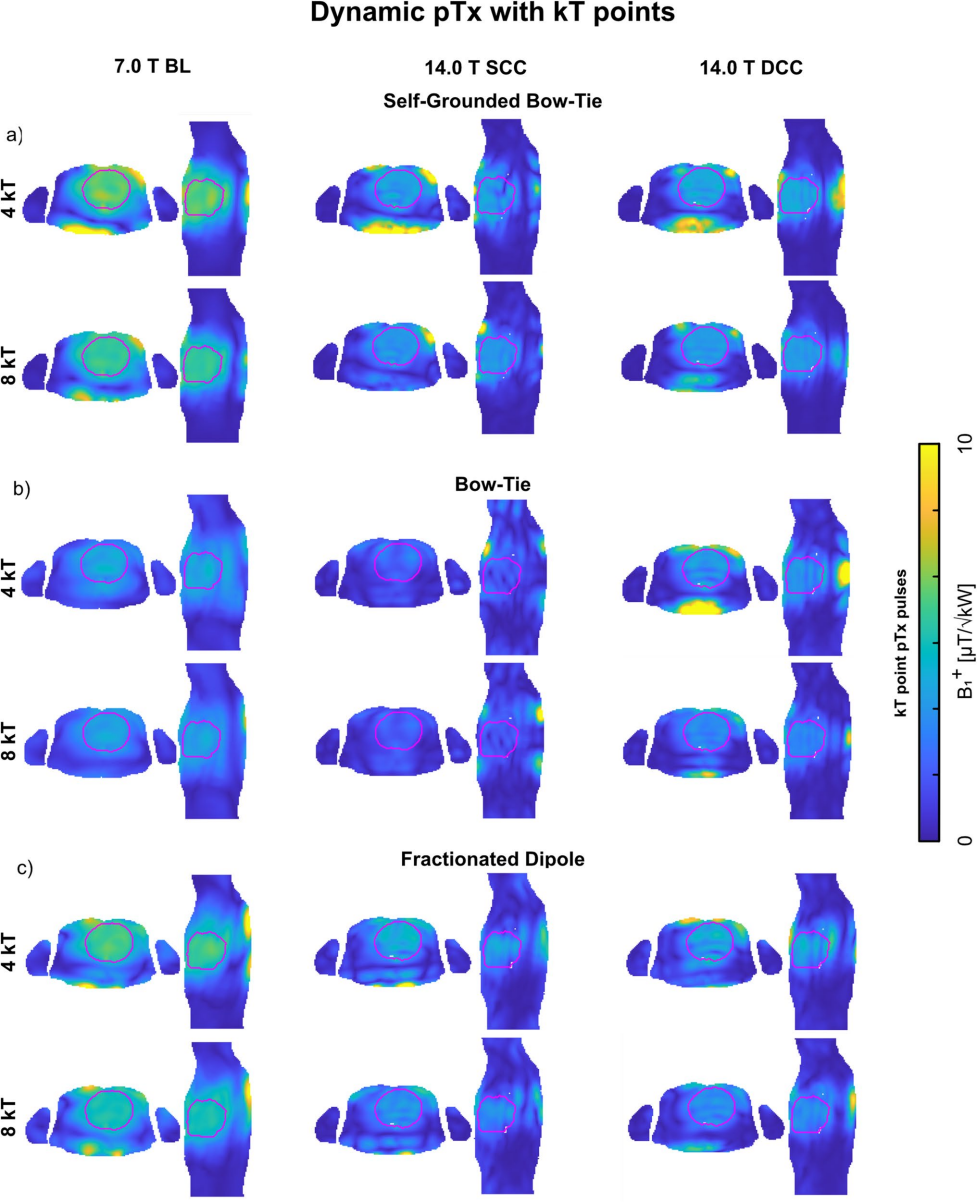
Axial and sagittal views through the center of the heart (depicted in red) showing the B_1_^+^ efficiency maps (B_1_^+^/√1 kW) using the **a** self-grounded bow-tie antenna building block; **b** bow-tie antenna building block; and c) the fractionated dipole antenna RF array configurations at 7.0 T (baseline, BL) and 14.0 T (same channel count, SCC, double channel count, DCC). Dynamic pTx was performed with tailored kT-points, using a series of RF sub-pulses and gradient blips to achieve a homogeneous flip angle (FA) within the 3D ROI targeting the heart. For the optimization, a nominal FA of 10° was targeted in the heart ROI by using 4 and 8 kT-points, with a total pulse duration of 0.96 ms and 1.92 ms, respectively. The FA maps of the pulse design were scaled into B_1_^+^ efficiency maps

Performing dynamic pTx (Fig. 6) with 4 kT points for Duke revealed for the 14.0 T SCC setups a worst-case CoV < 28% with minimum B_1_^+^_ROI_ < 0.56 µT/√kW, and maximum SAR_10g_ < 3.26 W/kg. The DCC setups with 4 kT points had lower CoV with enhanced minimum B_1_^+^_ROI_ and reduced SAR level (Table 6). Increasing to 8 kT points revealed a worst-case CoV < 20% at 14.0 T for the SCC setups, with minimum B_1_^+^_ROI_ < 0.59 µT/√kW and maximum SAR_10g_ < 8.15 W/kg (Table 6). The DCC setups with 8 kT points had lower CoV with enhanced minimum B_1_^+^_ROI_ and reduced SAR level (Table 6). The corresponding results for Ella are shown in Table 6.

**Table 6:** Summary of the mean B_1_^+^, minimum B_1_^+^, coefficient of variation (CoV(B_1_^+^_ROI_)) across the entire heart of the human voxel models Duke and Ella, and the maximum SAR_10g_ for 4 and 8 kT point pTx pulses using self-grounded bow-tie (SGBT) antenna building block, bow-tie (BT) antenna building block, and fractionated dipole (FD) antenna RF arrays at 7.0 T (baseline, BL) and 14.0 T (same channel count, SCC, double channel count, DCC). The total RF power for the excitation vectors (P_fwd_) is presented for a lossless 2 kW power at each channel

	Dynamic pTx using kT points on Duke				
	mean B_1_^+^_ROI_ [µT/√kW]	min. B_1_^+^_ROI_ [µT/√kW]	max. SAR_10g_ [W/kg]	CoV [%]	P_fwd_ [kW]
4 kT points					
7.0 T BL					
SGBT	6.10	4.66	1.36	6	11
BT	3.75	2.39	0.95	11	27
FD	5.56	3.85	0.85	7	13
14.0 T SCC					
SGBT	3.67	1.44	3.18	14	28
BT	2.03	0.56	3.26	28	66
FD	3.53	0.66	2.80	21	28
14.0 T DCC					
SGBT	3.76	1.80	1.69	13	52
BT	3.13	0.93	2.71	17	36
FD	3.41	0.63	1.33	22	30
8 kT points					
7.0 T BL					
SGBT	5.52	4.47	2.77	5	14
BT	3.59	2.53	1.90	8	31
FD	4.96	3.95	1.90	5	17
14.0 T SCC					
SGBT	3.27	1.51	6.07	10	36
BT	1.73	0.59	8.15	20	105
FD	3.00	1.17	5.13	15	42
14.0 T DCC					
SGBT	3.40	1.79	3.18	10	95
BT	2.81	1.23	5.25	12	48
FD	2.90	1.11	2.86	14	45

	Dynamic pTx using kT points on Ella				
	mean B_1_^+^_ROI_ [µT/√kW]	min. B_1_^+^_ROI_ [µT/√kW]	max. SAR_10g_ [W/kg]	CoV [%]	P_fwd_ [kW]
4 kT points					
7.0 T BL					
SGBT	7.30	5.71	1.19	6	8
BT	4.89	3.74	0.73	7	17
FD	7.57	5.85	0.91	9	7
14.0 T SCC					
SGBT	4.50	2.60	5.94	11	19
BT	2.65	1.04	2.65	23	44
FD	4.00	1.59	2.25	16	23
14.0 T DCC					
SGBT	4.03	2.18	2.00	11	23
BT	3.94	1.86	2.17	12	24
FD	4.29	1.57	1.69	20	20
8 kT points					
7.0 T BL					
SGBT	6.89	5.75	2.59	4	9
BT	4.46	3.55	1.53	5	20
FD	6.30	5.13	1.88	4	11
14.0 T SCC					
SGBT	4.12	2.79	9.87	8	24
BT	2.40	1.02	4.45	16	61
FD	3.73	2.01	3.89	12	28
14.0 T DCC					
SGBT	4.18	2.77	4.40	8	23
BT	3.66	2.05	4.24	10	29
FD	3.76	1.39	3.27	17	27

### Assessment of noise amplification (G-factor)

The assessment of the noise amplification due to PI for Duke is summarized in Table 7, which shows the mean and maximum *g*-factors of the RF arrays under investigation. Two-fold acceleration R_y_ along the main axis of the RF arrays (phase encoding along the L-R direction) revealed a maximum noise amplification of *g*_*max*_ = 1.04 and g_*max*_ < 2.79 with R_y_ = 4 for all RF arrays at 7.0 T BL. At 14.0 T, the SCC setups had *g*_*max*_ < 1.29 for two-fold acceleration, and for R_y_ = 4 a *g*_*max*_ < 3.28 was found. The DCC setup with increased channel count had reduced *g*_*max*_ < 1.06 for two-fold acceleration and R_y_ = 4 a *g*_*max*_ < 1.60 at 14.0 T. The corresponding noise amplification values along the A-P phase encoding direction (R_x_) are shown in Table 7.

**Table 7:** The (a) mean and (b) maximum g-factors of the self-grounded bow-tie (SGBT) antenna building block, bow-tie (BT) antenna building block, and the fractionated dipole (FD) antenna RF array configurations at 7.0 T (baseline, BL) and 14.0 T (same channel count, SCC, double channel count DCC) in the cardiac ROI of Duke for SENSE image reduction for R = 2–4. The g-factors are given in the anterior–posterior (R_x_) and left–right (R_y_) phase encoding direction

	Noise amplification								
	SGBT			BT			FD		
(a) mean	7.0 T	14.0 T	14.0 T^+^	7.0 T	14.0 T	14.0 T^+^	7.0 T	14.0 T	14.0 T^+^
R_y_ = 2	1.00	1.00	1.00	1.00	1.02	1.00	1.00	1.00	1.00
R_y_ = 3	1.02	1.02	1.00	1.06	1.10	1.01	1.02	1.04	1.00
R_y_ = 4	1.09	1.02	1.02	1.26	1.23	1.06	1.06	1.06	1.02
R_x_ = 2	1.02	1.01	1.01	1.05	1.02	1.03	1.04	1.04	1.02
R_x_ = 3	1.26	1.11	1.11	1.85	1.27	1.30	1.53	1.47	1.18
R_x_ = 4	1.49	1.25	1.27	2.61	1.54	1.65	1.91	1.81	1.35
(b) max	7.0 T	14.0 T	14.0 T^+^	7.0 T	14.0 T	14.0 T^+^	7.0 T	14.0 T	14.0 T^+^
R_y_ = 2	1.04	1.05	1.01	1.04	1.29	1.06	1.04	1.09	1.01
R_y_ = 3	1.21	1.49	1.06	1.39	2.44	1.18	1.15	1.36	1.14
R_y_ = 4	1.67	1.31	1.11	2.39	3.28	1.60	2.79	2.90	1.39
R_x_ = 2	1.16	1.17	1.13	1.46	1.59	1.43	1.30	1.66	1.24
R_x_ = 3	1.87	1.57	1.92	4.22	3.77	2.41	7.99	4.08	1.70
R_x_ = 4	2.64	2.26	2.58	7.94	4.83	3.70	15.41	5.97	2.19

The DCC setups at 14.0 T are indicated with ^+^

## Discussion

This work examines the electromagnetic challenges of CMR at 14.0 T, and provides RF coil concepts that address the electrodynamic constraints of imaging the human heart at 14.0 T based on EMF simulations. Our numerical findings indicate that CMR at 14.0 T is feasible with realistic RF antenna systems, and provides a foundation for further exploration and real-world implementation. This simulation study presents results derived from the human voxel models Duke and Ella. The larger upper torso and cardiac ROI of Duke as compared to the female human voxel model Ella makes the male model more challenging for CMR, with lower B_1_^+^ efficiency and homogeneity. Here we focus on the male voxel model Duke, given the more challenging application and for the reason that both voxel models showed similar behavior at 14.0 T CMR. Furthermore, the antennas were designed for 7.0 T MR application and are not optimized antenna designs for 14.0 T CMR. For simplicity the antenna dimensions were scaled linearly to the magnetic field strength, resulting in undesired losses in the antenna. However, it has been shown that electrodynamic scaling is a feasible approach for investigating RF behavior at varying static magnetic field strengths [87]. Furthermore, losses in the signal chain, or resulting from cardiac motion were not considered in this study.

From the co-simulation sufficient tuning and matching were obtained with neglectable losses. The SGBT and FD arrays revealed a model-specific tuning and matching network, whereas the BT array showed a robust network against different body models. Such a model-specific tuning and matching network would indeed make a real-life application more challenging, and a trade-off between the tuning and matching network of the different body types would be necessary and would result in higher worst-case reflection and coupling. This would lead to increased losses.

The shortened antennas of the SCC setups resulted in a narrower FOV of the antenna. The narrow FOV and the larger distance between the BBs at 14.0 T caused less interference of the individual EMFs. Along with the higher losses at 14.0 T, this resulted in a lower TXE and iSNR compared to the 7.0 T BL setups. The wavelength and antenna shortening at 14.0 T improved the antenna density per unit area, allowing for twice the number of BBs for the DCC setups. The enhanced channel density of the DCC setup is beneficial to offset the reduction of B_1_^+^ and B_1_^−^ superposition. The enhanced density of the DCC setups and the closer-positioned antennas allowed better control of the EMFs. The intrinsic B_1_^+^ and B_1_^−^ superposition yielded higher mean TXE and iSNR for the DCC setups (14.0 T) compared to the SCC setups (14.0 T) and the 7.0 T baseline setups. This is because the higher channel count enabled a greater degree of freedom. However, a TXE and iSNR gradient between the periphery and the center of the body was obtained. For the latter, minimum TXE and iSNR remained below the minimum obtained for the 7.0 T BL setups. This behavior was already reported at lower field strength [88] and remains a major constraint and challenge of CMR. At 14.0 T the performance ratio of the three RF array concepts showed an increase of < 8% losses in the antenna and coupling compared to the 7.0 T baseline setups. This difference suggests that the electrodynamic scaling of the antennas is feasible, with only a minor impact on the transmit/receive performance. The SGBT array at 14.0 T had values almost twice as high for TXE and iSNR compared to the BT (high losses) and compared to the FD (4 × lower channel count). To achieve the enhanced TXE and iSNR values, the SGBT array with enhanced channel count will require more total RF power. This is also reflected in the total RF power obtained from the static and dynamic pTx optimization.

Enlarging the number of BBs is conceptually appealing to increase the degrees of freedom for B_1_^+^ shaping and uniform B_1_^+^ distribution, as seen for the optimal B_1_ superposition. At 7.0 T, phase-optimized pTx provided sufficient performance to reduce B_1_^+^ efficiency (Eq. 2) and inhomogeneity (Eq. 3) across the whole 3D heart. At 14.0 T phase optimized pTx targeting the whole 3D heart showed limitations, while phase and amplitude optimized pTx showed promising results with maximized minimum B_1_^+^_ROI_ < 1.01 µT/√kW (Duke) for the SGBT SCC setup, which was approximately twice the minimum B_1_^+^_ROI_ of the BT and FD RF arrays. The higher minimum B_1_^+^_ROI_ of the SGBT array is reflected on the B_1_^+^ superposition. The higher minimum B_1_^+^_ROI_ of the SGBT comes with an elevated SAR level (7.01 W/kg), which resulted in the lowest SAR efficiency (mean B_1_^+^/√SAR) of the three concepts, while the FD showed the highest SAR efficiency. The increased channel count of the DCC setups resulted in greater B_1_^+^ efficiency and reduced maximum SAR_10g_, with optimized minimum B_1_^+^_ROI_ compared to the SCC setups, resulting in greater SAR efficiency (< + 20%). The higher SAR efficiency yielded less RF input power consumption to achieve an equivalent FA while staying within the safety limits [89].

To more closely examine RF power deposition with respect to safety requirements [89], we included the objective of SAR_10g_ in our optimizations. MOO offers options for a trade-off between the objective of minimum B_1_^+^_ROI_ and the objective of maximum SAR_10g_. Phase-optimized pTx showed limited performance with respect to an optimized SAR efficiency (< − 3%). Phase and amplitude-optimized pTx MOO enabled a decreased SAR level (< − 88%) with only a minor reduction in minimum B_1_^+^_ROI_ (< − 29%), resulting in enhanced SAR efficiency (< + 117%), which underlines the value of the MOO approach at 14.0 T. The results for Ella showed similar behavior with only higher B_1_^+^ efficiency values for the static pTx approach.

The static pTx approach provided limited performance at 14.0 T where no signal dropouts were obtained, but the challenges of transmission inhomogeneity could not be fully addressed. Approaching this obstacle, we performed the CoV optimization (Eq. 3) but the results were not promising. Including Eq. 3 as one of the objectives in the MOO yielded insufficient results where the DCC setups had CoV > 29% with a SAR level < 0.63 W/kg and a minimum B_1_^+^_ROI_ < 0.27 µT/√kW. To tackle these challenges, the dynamic pTx using kT-points was performed. The scaled B_1_^+^ maps with dynamic pTx revealed a more uniform B_1_^+^ distribution compared to the static pTx approach with optimized CoV. However, the improved CoV was associated with reduced B_1_^+^ efficiency. Increasing the number of sub-RF-pulses showed an improved CoV, but with a more enhanced SAR level which is a major safety concern. Increasing the channel count for the DCC setups could address this obstacle with lower CoV as well as lower SAR level compared to the SCC setups. Dynamic pTx with 8 kT points in conjunction with the increased channel density of the DCC setups showed the best results for the SGBT RF array, with improved CoV (10%) compared to the static pTx (26%) at 14.0 T, while achieving a minimum B_1_^+^_ROI_ = 1.79 µT/√kW and a maximum SAR_10g_ < 3.18 W/kg. The higher degrees of freedom of the dynamic pTx approach will require more total RF power than the static pTx approach. These results obtained from the dynamic pTx using the DCC setups at 14.0 T are competitive when benchmarked against previous reports on CMR at 3.0 T and 7.0 T. For CMR at 3.0 T a CoV of 31% was reported for cardiac ROI covering the whole heart [88, 90, 91]. Dynamic pTx at 7.0 T using 4 kT points yielded a CoV of ~ 10% [52].

Our assessment of the parallel imaging performance of CMR at 7.0 T and 14.0 T confirmed previous reports that showed reduced noise amplification at higher magnetic field strengths for an elliptic cylinder or a sphere, using magnetic field strengths up to 11.5 T [67]. Parallel acquisition of the upper torso and the use of higher magnetic field strengths are synergistic because with the wavelength shortening PI becomes more effective in large objects. This advantage facilitates higher acceleration factors for CMR at 14.0 T compared to 7.0 T. This PI gain would benefit CMR in the presence of physiological motion, and further real-time imaging of the heart. By doubling the Rx channel count, the DCC setups at 14.0 T led to a reduction in the mean and maximum *g*-factors compared to the SCC configurations and the 7.0 T baseline setups. The DCC setup of the SGBT RF array showed the best PI performance. The improved PI performance at higher magnetic field strengths can be further enhanced by increasing the channel count, as previously demonstrated for accelerated cardiac MRI at 3.0 T [92, 93].

Our results indicate that a multi-transmit system beyond the current state-of-the-art 8 or 16 Tx channels will be essential for CMR at 14.0 T. The literature shows that pTx systems with > 16 Tx channels are very feasible [71, 94]. Increasing the Tx channel count would further improve B_1_^+^ efficiency, homogeneity, and SAR efficiency. The limiting factors for enhanced channel density are the dimensions of the Tx elements, as well as the coupling because the anatomical coverage is limited on the upper torso. The low coupling and compact size of the SGBT BB allowed up to 64 elements (14.0 T) on the upper torso in the current study.

To summarize, of the three RF array configurations investigated, the SGBT array had the highest TXE and iSNR. The superior performance of the SGBT RF array configuration is due to the greater channel count per unit area compared to the BT (2x) and FD (4x) RF arrays, as well as the improved coupling of the EMF afforded by the dielectric pad. The higher channel count will require more total RF power in order the achieve the results presented. Nevertheless, the higher B_1_^+^ efficiency comes with an increased SAR level which might constitute an RF power deposition concern. This constraint of the SGBT array configuration was addressed by including SAR in the MOO. Using this approach, the SAR level obtained for phase and amplitude optimized pTx strategy of the SGBT was reduced by a factor of ~ 5.5 (0.77 W/kg versus 4.24 W/kg) while a minimum B_1_^+^_ROI_ of 0.73 µT/√kW (before 0.91 µT/√kW) was achieved. The dynamic pTx approach using kT points showed promising results where a uniform B_1_^+^ distribution could be achieved with increased kT points. This will also require more total RF power compared to the static pTx approach. The merits of the SGBT array configuration are not limited to the transmission side, but also yield enhanced coil sensitivity for reception versus the BT and the FD array configurations [95]. The 14.0 T DCC setup and the SGBT RF array were synergistic, and showed the best parallel imaging performance of the three RF coil configurations investigated.

## Conclusions 

While the number of reports on experimental and clinical research for cardiac and body UHF-MR at 7.0 T continues to grow, the first steps into the exploration of even higher magnetic field strengths are already being taken. While novel magnet technology will surely support MR at B_0_ > 11.7 T in the future, its use for cardiac MRI might be constrained by technical challenges, physiological limitations, and practical obstacles. These include the need for a better understanding of electrodynamic constraints that arise through increased spin excitation frequency. Power losses due to frequency-dependent changes in the conductive properties of tissues will occur, and several legitimate challenges concerning RF power deposition restrictions, B_1_^+^ efficiency constraints, depth penetration limitations, and radiation losses will need to be resolved. These challenges notwithstanding, this study indicates that an MRI of the human heart at 14.0 T is feasible from an electrodynamic and theoretical standpoint. These findings open the door to further research that might catalyze a next-generation 14.0 T human MR system. Such systems will undoubtedly unveil new dimensions of the processes of cardiac health and disease.

## Supplementary Information

Below is the link to the electronic supplementary material.

Supplementary file1 (DOCX 3894 KB)

## Data Availability

The antenna models and the cardiac RF arrays on the human voxel models from CST Studio Suite 2020 can be downloaded from https://github.com/bnurzed/Dipole-RF-Arrays-for-cardiac-MRI-. The code for the kT point pulse design can be downloaded from https://github.com/chaigner/UP_body.
