# Supplementary material for: Radiofrequency antenna concepts for human cardiac MR at 14.0 T
Source: MAGMA. 2023 Mar 15;36(2):257–77. doi: 10.1007/s10334-023-01075-1 (PMC10140016; doi:10.1007/s10334-023-01075-1)
Supplement: Supplementary file 1 — Supplementary file1 (DOCX 3894 KB) [file 10334_2023_1075_MOESM1_ESM.docx]

# Supplementary


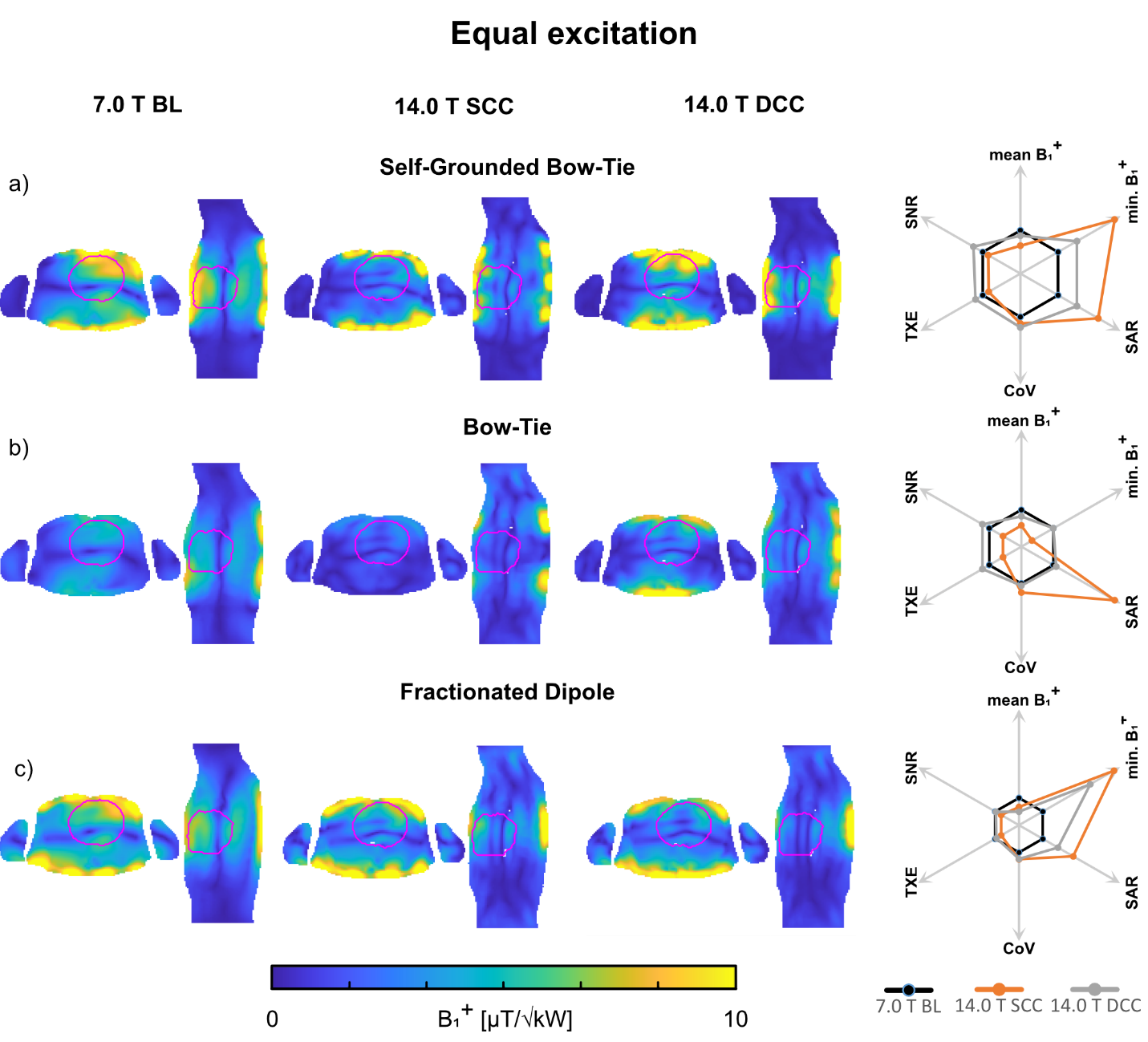
Figure S1: Axial and sagittal views through the center of the heart showing B_1_^+^ efficiency maps (B_1_^+^ /√1kW) obtained for an equal phase (0) and equal amplitude (1) excitation using the a) self-grounded bow-tie antenna building block; b) bow-tie antenna building block; and c) the fractionated dipole antenna RF array configurations at 7.0 T (baseline, BL) and 14.0 T (same channel count, SCC, double channel count, DCC). The cardiac ROI is depicted in red. The spider diagrams illustrate the relative changes of the mean B_1_^+^_ROI_, minimum B_1_^+^_ROI_, maximum SAR_10g_, CoV(B_1_^+^_ROI_), TXE, and intrinsic SNR values for the 14.0 T SCC (orange) and DCC (grey) with respect to the 7.0 T baseline(black).

Table S1 Summary of the mean B_1_^+^, minimum B_1_^+^ , coefficient of variation (CoV(B_1_^+^_ROI_)) across the entire heart of the human voxel models Duke and Ella, and the maximum SAR_10g_ for an excitation vector with equal phase (0°) and amplitude (1) for all channels using self-grounded bow-tie (SGBT) antenna building block, bow-tie (BT) antenna building block, and fractionated dipole (FD) antenna RF arrays at 7.0 T (baseline, BL) and 14.0 T (same channel count, SCC, double channel count, DCC). The total RF power for the excitation vectors (P_fwd_) is presented for a lossless 2kW power at each channel.

| **Excitation with equal phase (0°) and amplitude (1)** | | | | | | |
| --- | --- | --- | --- | --- | --- | --- |
| **Duke** | | mean B_1_^+^_ROI_ [µT/√kW] | min. B_1_^+^_ROI_  [µT/√kW] | max. SAR_10g_ [W/kg] | CoV  [%] | P_fwd_ [kW] |
| 7.0 T BL | SGBT | 5.21 | 0.02 | 0.30 | 45 | 64 |
|  | BT | 3.40 | 0.03 | 0.23 | 44 | 32 |
|  | FD | 5.05 | 0.01 | 0.25 | 39 | 16 |
| 14.0 T SCC | SGBT | 3.37 | **0.05** | 0.62 | 52 | 64 |
|  | BT | 1.96 | 0.01 | **0.67** | 55 | 32 |
|  | FD | 3.38 | 0.04 | 0.57 | 48 | 16 |
| 14.0 T DCC | SGBT | 4.58 | 0.03 | 0.45 | **56** | 128 |
|  | BT | 2.80 | 0.03 | 0.25 | 46 | 64 |
|  | FD | 2.51 | 0.03 | 0.41 | 48 | 32 |
| **Ella** |  |  |  |  |  |  |
| 7.0 T BL | SGBT | 5.41 | **0.06** | 0.29 | 39 | 64 |
|  | BT | 4.31 | 0.04 | 0.19 | 41 | 32 |
|  | FD | 6.17 | **0.06** | 0.28 | 39 | 16 |
| 14.0 T SCC | SGBT | 3.70 | 0.01 | **1.08** | 51 | 64 |
|  | BT | 2.44 | 0.02 | 0.50 | 44 | 32 |
|  | FD | 3.51 | 0.02 | 0.40 | 41 | 16 |
| 14.0 T DCC | SGBT | 4.85 | 0.03 | 0.49 | **52** | 128 |
|  | BT | 3.21 | 0.04 | 0.22 | 39 | 64 |
|  | FD | 2.79 | 0.03 | 0.27 | 42 | 32 |


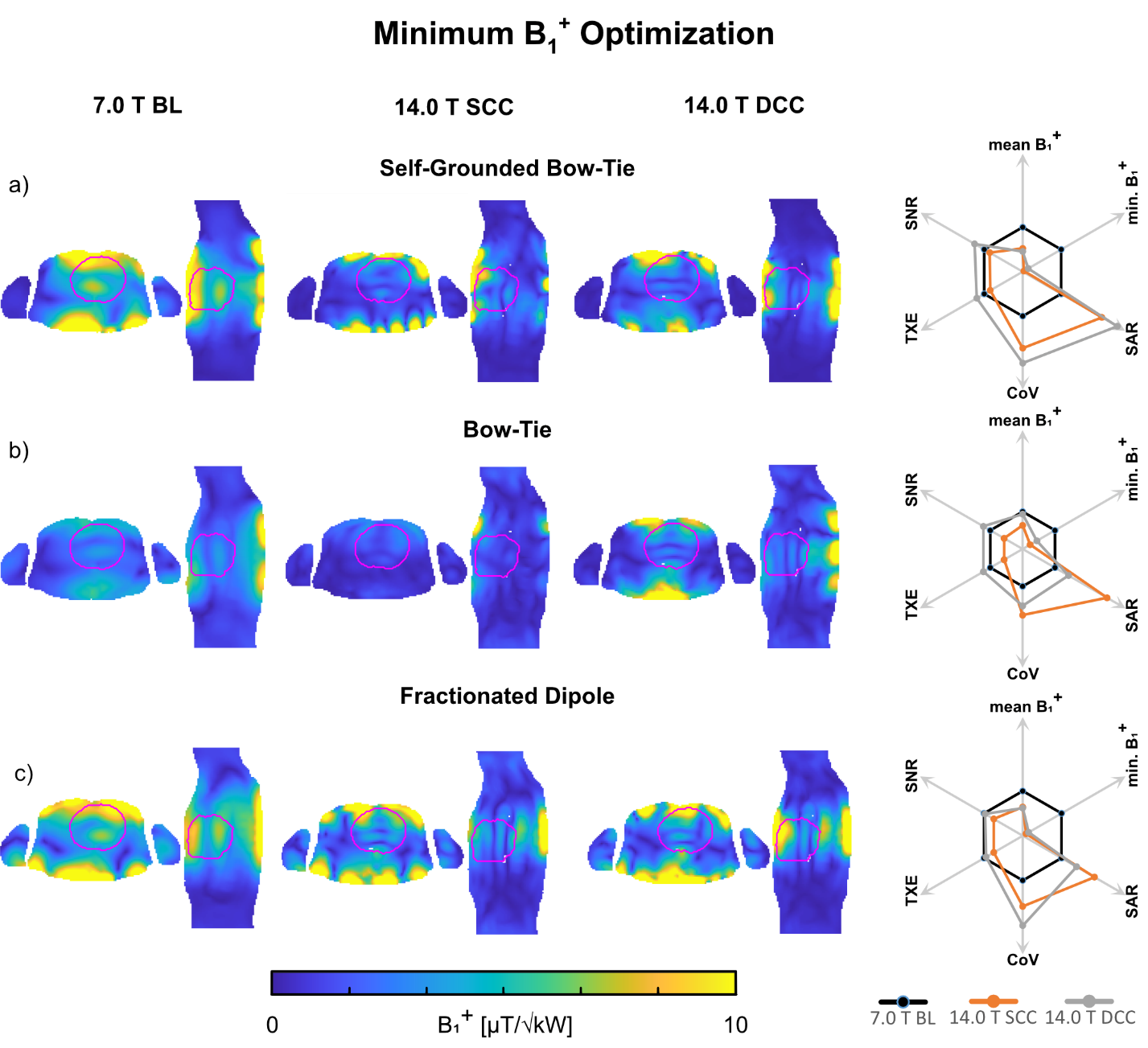
Figure S2: Axial and sagittal views through the center of the heart showing B_1_^+^ efficiency maps (B_1_^+^ /√1kW) obtained for static pTx phase shimming using the a) self-grounded bow-tie antenna building block; b) bow-tie antenna building block; and c) the fractionated dipole antenna RF array configurations at 7.0 T (baseline, BL) and 14.0 T (same channel count, SCC, double channel count, DCC). The cardiac ROI is depicted in red. The superposed minimum B_1_^+^ of all channels within the whole 3D cardiac ROI was maximized in the optimization process. The spider diagrams illustrate the relative changes of the mean B_1_^+^_ROI_, minimum B_1_^+^_ROI_, maximum SAR_10g_, CoV(B_1_^+^_ROI_), TXE, and intrinsic SNR values for the 14.0 T SCC (orange) and DCC (grey) with respect to the 7.0 T baseline(black).

Table S2: Summary of the mean B_1_^+^, minimum B_1_^+^, coefficient of variation (CoV(B_1_^+^_ROI_)) across the entire heart of the human voxel models Duke and Ella, and the maximum SAR_10g_ for a phase pTx approach with optimized minimum B_1_^+^ in the ROI self-grounded bow-tie (SGBT) antenna building block, bow-tie (BT) antenna building block, and fractionated dipole (FD) antenna RF arrays at 7.0 T (baseline, BL) and 14.0 T (same channel count, SCC, double channel count, DCC). The total RF power for the excitation vectors (P_fwd_) is presented for a lossless 2kW power at each channel.

| **Minimum B_1_^+^ Optimization** | | | | | | |
| --- | --- | --- | --- | --- | --- | --- |
| **Duke** | | mean B_1_^+^_ROI_ [µT/√kW] | **min. B_1_^+^_ROI_**  **[µT/√kW]** | max. SAR_10g_ [W/kg] | CoV  [%] | P_fwd_ [kW] |
| 7.0 T BL | SGBT | 5.85 | **3.16** | 0.35 | 34 | 64 |
|  | BT | 2.87 | **1.46** | 0.31 | 34 | 32 |
|  | FD | 5.49 | **2.57** | 0.41 | 32 | 16 |
| 14.0 T SCC | SGBT | 3.06 | **0.10** | 0.72 | 59 | 64 |
|  | BT | 1.82 | **0.34** | 0.81 | 60 | 32 |
|  | FD | 3.53 | **0.22** | 0.76 | 51 | 16 |
| 14.0 T DCC | SGBT | 2.66 | **0.45** | 0.86 | 70 | 128 |
|  | BT | 2.68 | **0.64** | 0.44 | 51 | 64 |
|  | FD | 3.39 | **0.39** | 0.57 | 65 | 32 |
| **Ella** |  |  |  |  |  |  |
| 7.0 T BL | SGBT | 6.69 | **4.17** | 0.35 | 24 | 64 |
|  | BT | 3.71 | **1.93** | 0.34 | 27 | 32 |
|  | FD | 6.99 | **3.71** | 0.36 | 25 | 16 |
| 14.0 T SCC | SGBT | 4.01 | **1.12** | 0.81 | 54 | 64 |
|  | BT | 2.12 | **0.58** | 0.50 | 42 | 32 |
|  | FD | 4.51 | **0.52** | 0.60 | 55 | 16 |
| 14.0 T DCC | SGBT | 4.65 | **1.33** | 0.77 | 52 | 128 |
|  | BT | 2.87 | **0.89** | 0.54 | 37 | 64 |
|  | FD | 3.47 | **0.69** | 0.72 | 50 | 32 |


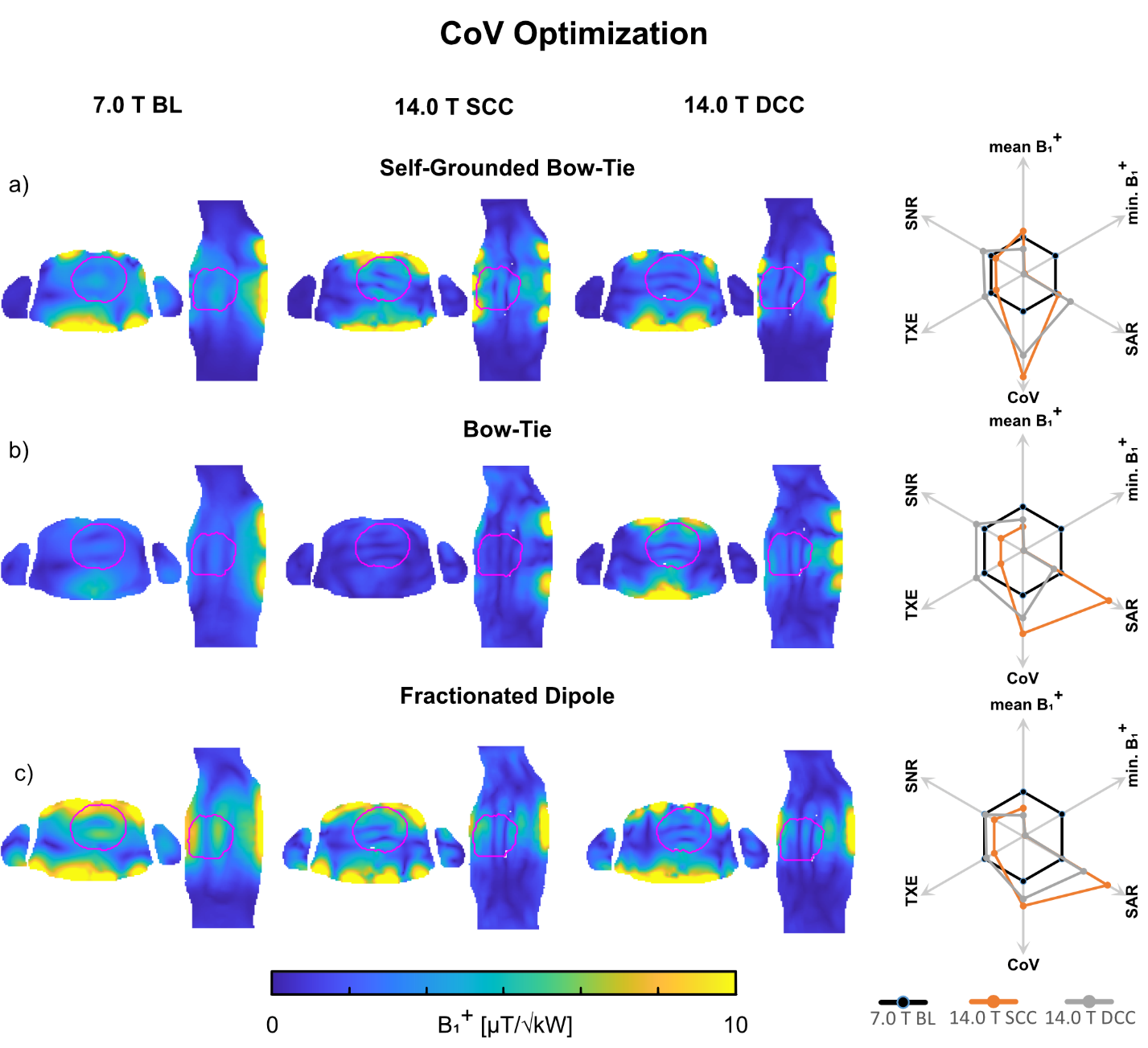
Figure S3: Axial and sagittal views through the center of the heart showing B_1_^+^ efficiency maps (B_1_^+^ /√1kW) obtained for static pTx phase shimming using the a) self-grounded bow-tie antenna building block; b) bow-tie antenna building block; and c) the fractionated dipole antenna RF array configurations at 7.0 T (baseline, BL) and 14.0 T (same channel count, SCC, double channel count, DCC). The cardiac ROI is depicted in red. The CoV (B_1_^+^) within the whole 3D cardiac ROI was minimized in the optimization process. The spider diagrams illustrate the relative changes of the mean B_1_^+^_ROI_, minimum B_1_^+^_ROI_, maximum SAR_10g_, CoV(B_1_^+^_ROI_), TXE, and intrinsic SNR values for the 14.0 T SCC (orange) and DCC (grey) with respect to the 7.0 T baseline(black). The total RF power for the excitation vectors (P_fwd_) is presented for a lossless 2kW power at each channel.

Table S3: Summary of the mean B_1_^+^, minimum B_1_^+^, coefficient of variation (CoV(B_1_^+^_ROI_)) across the entire 3D heart of the human voxel models Duke and Ella, and the maximum SAR_10g_ for a phase pTx approach with optimized CoV(B_1_^+^_ROI_) in the ROI using self-grounded bow-tie (SGBT) antenna building block, bow-tie (BT) antenna building block, and fractionated dipole (FD) antenna RF arrays at 7.0 T (baseline, BL) and 14.0 T (same channel count, SCC, double channel count, DCC). The total RF power for the excitation vectors (P_fwd_) is presented for a lossless 2kW power at each channel.

| **CoV Optimization** | | | | | | |
| --- | --- | --- | --- | --- | --- | --- |
| **Duke** | | mean B_1_^+^_ROI_ [µT/√kW] | min. B_1_^+^_ROI_  [µT/√kW] | max. SAR_10g_ [W/kg] | **CoV**  **[%]** | P_fwd_ [kW] |
| 7.0 T BL | SGBT | 2.99 | 1.06 | 0.56 | **15** | 64 |
|  | BT | 1.93 | 0.75 | 0.37 | **21** | 32 |
|  | FD | 5.29 | 1.70 | 0.34 | **28** | 16 |
| 14.0 T SCC | SGBT | 3.49 | 0.04 | 0.61 | **42** | 64 |
|  | BT | 1.06 | 0.02 | 0.83 | **40** | 32 |
|  | FD | 3.36 | 0.08 | 0.74 | **44** | 16 |
| 14.0 T DCC | SGBT | 2.04 | 0.02 | 0.82 | **33** | 128 |
|  | BT | 1.37 | 0.02 | 0.30 | **32** | 64 |
|  | FD | 2.50 | 0.09 | 0.53 | **40** | 32 |
| **Ella** |  |  |  |  |  |  |
| 7.0 T BL | SGBT | 4.31 | 2.16 | 0.78 | **15** | 64 |
|  | BT | 2.73 | 0.87 | 0.21 | **19** | 32 |
|  | FD | 6.25 | 2.02 | 0.43 | **21** | 16 |
| 14.0 T SCC | SGBT | 2.77 | 0.06 | 0.95 | **34** | 64 |
|  | BT | 1.41 | 0.02 | 0.49 | **33** | 32 |
|  | FD | 3.69 | 0.08 | 0.64 | **35** | 16 |
| 14.0 T DCC | SGBT | 1.93 | 0.54 | 0.81 | **28** | 128 |
|  | BT | 2.00 | 0.08 | 0.56 | **27** | 64 |
|  | FD | 3.19 | 0.01 | 0.62 | **34** | 32 |


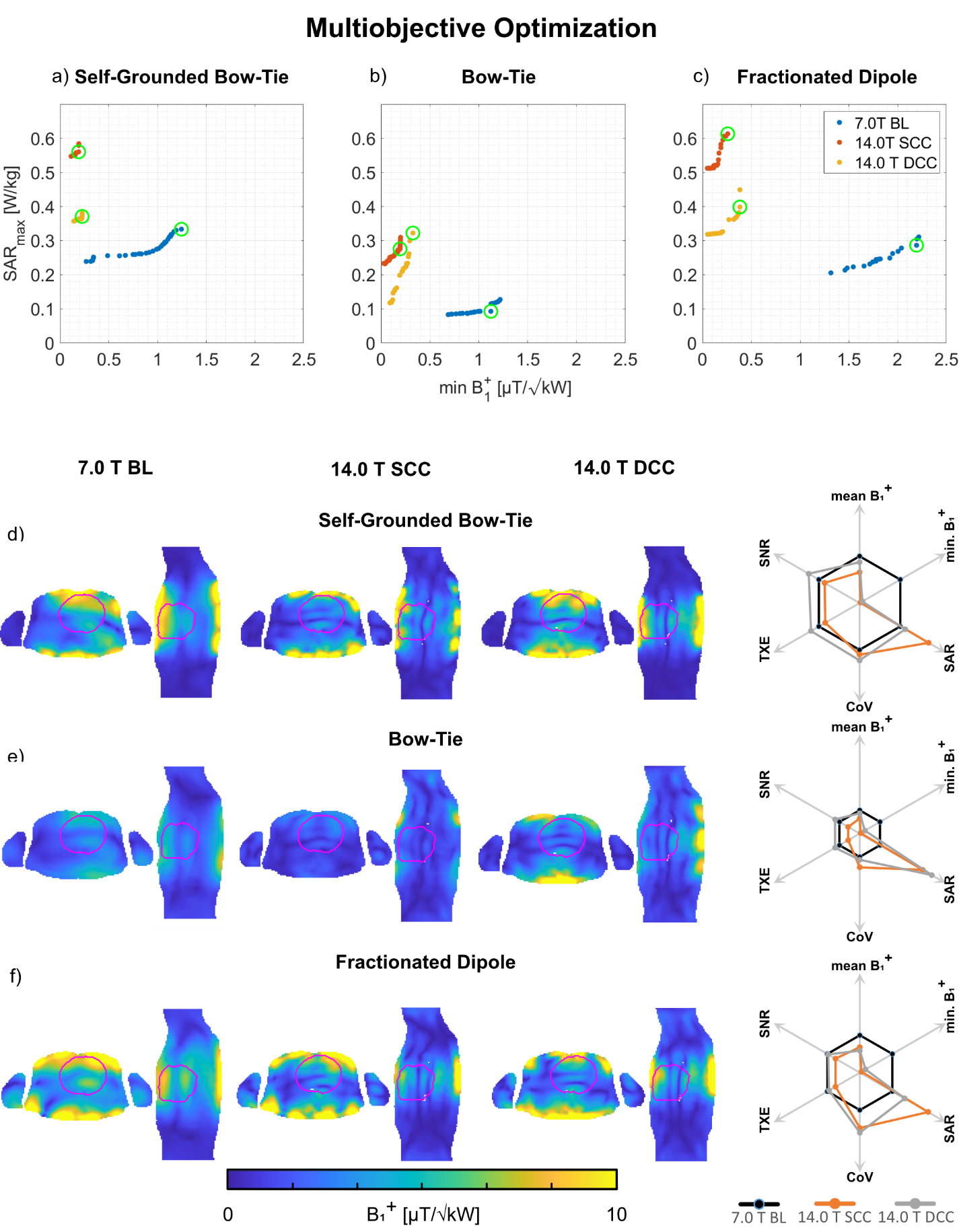
Figure S4: a-c) Pareto front derived from the static phase optimized pTx MOO approach using the a) self-grounded bow-tie antenna building block; b) bow-tie antenna building block; and c) the fractionated dipole antenna RF array configurations at 7.0 T (baseline, BL) and 14.0 T (same channel count, SCC, double channel count, DCC). Each point of the solution represents one optimized excitation vector where a trade-off between the minimum B_1_^+^_ROI_ and the maximum SAR_10g_ was found. The green circles indicate the highest minimum B_1_^+^_ROI_/√SAR_10g_ ratio d-f): Axial and sagittal views through the center of the heart (depicted in red) illustrating B_1_^+^ efficiency maps (B_1_^+^ /√1kW) obtained for the excitation vectors with highest minimum B_1_^+^_ROI_/√SAR_10g_ ratio (indicated by the green circles in a-c). The spider diagrams illustrate the relative changes of the mean B_1_^+^_ROI_, minimum B_1_^+^_ROI_, maximum SAR_10g_, CoV(B_1_^+^_ROI_), TXE, and intrinsic SNR values for the 14.0 T SCC (orange) and DCC (grey) with respect to the 7.0 T baseline (black).

Table S4: Summary of the mean B_1_^+^, minimum B_1_^+^, coefficient of variation (CoV(B_1_^+^)) across the entire 3D heart of the human voxel models Duke and Ella, and the maximum SAR_10g_ for the multiobjective phase optimizer with a trade-off between minimum B_1_^+^_ROI_ in the heart and maximum SAR_10g_ using self-grounded bow-tie (SGBT) antenna building block, bow-tie (BT) antenna building, and fractionated dipole (FD) antenna RF arrays at 7.0 T (baseline, BL) and 14.0 T (same channel count, SCC, double channel count, DCC). The total RF power for the excitation vectors (P_fwd_) is presented for a lossless 2kW power at each channel.

| **Multiobjective Optimization** | | | | | | |
| --- | --- | --- | --- | --- | --- | --- |
| **Duke** | | mean B_1_^+^_ROI_ [µT/√kW] | **min. B_1_^+^_ROI_**  **[µT/√kW]** | **max. SAR_10g_ [W/kg]** | CoV  [%] | P_fwd_ [kW] |
| 7.0 T BL | SGBT | 5.17 | **1.17** | **0.33** | 44 | 64 |
|  | BT | 2.98 | **1.10** | **0.09** | 40 | 32 |
|  | FD | 5.41 | **2.19** | **0.29** | 34 | 16 |
| 14.0 T SCC | SGBT | 3.39 | **0.03** | **0.56** | 49 | 64 |
|  | BT | 1.94 | **0.05** | **0.28** | 58 | 32 |
|  | FD | 3.68 | **0.11** | **0.61** | 50 | 16 |
| 14.0 T DCC | SGBT | 4.50 | **0.08** | **0.37** | 54 | 128 |
|  | BT | 2.59 | **0.28** | **0.32** | 45 | 64 |
|  | FD | 3.19 | **0.38** | **0.40** | 55 | 32 |
| **Ella** |  |  |  |  |  |  |
| 7.0 T BL | SGBT | 5.45 | **2.10** | **0.35** | 34 | 64 |
|  | BT | 3.09 | **1.11** | **0.16** | 34 | 32 |
|  | FD | 6.93 | **3.40** | **0.27** | 26 | 16 |
| 14.0 T SCC | SGBT | 3.48 | **0.06** | **0.51** | 48 | 64 |
|  | BT | 2.31 | **0.36** | **0.34** | 41 | 32 |
|  | FD | 3.83 | **0.39** | **0.52** | 53 | 16 |
| 14.0 T DCC | SGBT | 4.74 | **0.04** | **0.43** | 53 | 128 |
|  | BT | 3.14 | **0.04** | **0.19** | 41 | 64 |
|  | FD | 3.06 | **0.11** | **0.27** | 49 | 32 |
